# Searching for fat tails in CRISPR-Cas systems: Data analysis and mathematical modeling

**DOI:** 10.1371/journal.pcbi.1008841

**Published:** 2021-03-26

**Authors:** Yekaterina S. Pavlova, David Paez-Espino, Andrew Yu. Morozov, Ilya S. Belalov

**Affiliations:** 1 Mathematics Department, Palomar College, San Marcos, California, United States of America; 2 Department of Energy, Joint Genome Institute, Walnut Creek, California, United States of America; 3 Mammoth BioSciences, South San Francisco, California, United States of America; 4 School of Mathematics and Actuarial Science, University of Leicester, Leicester, United Kingdom; 5 Institute of Ecology and Evolution, Russian Academy of Sciences, Moscow, Russia; 6 Laboratory of Microbial Viruses, Winogradsky Institute of Microbiology, Research Center of Biotechnology RAS, Moscow, Russia; Rice University, UNITED STATES

## Abstract

Understanding CRISPR-Cas systems—the adaptive defence mechanism that about half of bacterial species and most of archaea use to neutralise viral attacks—is important for explaining the biodiversity observed in the microbial world as well as for editing animal and plant genomes effectively. The CRISPR-Cas system learns from previous viral infections and integrates small pieces from phage genomes called spacers into the microbial genome. The resulting library of spacers collected in CRISPR arrays is then compared with the DNA of potential invaders. One of the most intriguing and least well understood questions about CRISPR-Cas systems is the distribution of spacers across the microbial population. Here, using empirical data, we show that the global distribution of spacer numbers in CRISPR arrays across multiple biomes worldwide typically exhibits scale-invariant power law behaviour, and the standard deviation is greater than the sample mean. We develop a mathematical model of spacer loss and acquisition dynamics which fits observed data from almost four thousand metagenomes well. In analogy to the classical ‘rich-get-richer’ mechanism of power law emergence, the rate of spacer acquisition is proportional to the CRISPR array size, which allows a small proportion of CRISPRs within the population to possess a significant number of spacers. Our study provides an alternative explanation for the rarity of all-resistant super microbes in nature and why proliferation of phages can be highly successful despite the effectiveness of CRISPR-Cas systems.

## Author summary

About half of bacterial species and most of archaea are equipped with CRISPR-Cas systems of adaptive immunity to protect them from their natural enemies—bacteriophages. The memory of CRISPR-Cas contains a catalogue of the fingerprints of previously experienced offenders which is passed down to the bacterial progeny. The microbial resistance to viruses largely depends on the number of records in this CRISPR array. Our analysis combining metagenomics data and mathematical modelling shows that the size of CRISPR arrays in microbial populations generally follows a power law distribution. Power law distributions have been found in many other complex systems (earthquakes, financial markets, animal movement). We argue that our model explains the presence of a power law in CRISPR arrays and the rareness of all-resistant super microbes.

## Introduction

The abundance of bacterial and archaeal populations in natural and anthropogenic environments is largely controlled by their natural enemies—bacteriophages. Over the course of long-term evolution, however, prokaryotes have developed a number of efficient defence mechanisms, among which is the adaptive immunity system CRISPR-Cas (about half of bacterial species and most of archaea use CRISPR-Cas systems [1]). The CRISPR-Cas system learns from previous phage infections and passes down this information to subsequent bacterial generations [2, 3]. The most important current application of CRISPR-Cas systems is the genetic engineering of mammals and plants [4], with other applications including the release of modified CRISPR-Cas systems in natural and artificial environments [5–7]. A good understanding of the effect of CRISPR on the abundance and diversity of microbial populations as well as the consequences of the release of genetically modified bacteria into the environment can be only achieved *via* an interdisciplinary approach combining experimental work, mathematical modelling and extensive data analysis [8–12].

The CRISPR-Cas immune system contains a library of pieces of viral DNA (called spacers) originating from previous attempts to infect the microbial organism [13]. Spacers are separated by short identical sequences called the repeats. After transcription to RNA molecules (crRNA) and processing, spacers in the form of crRNA are bound to the CRISPR associated (Cas) effector proteins. In a second infection event from the same lineage of phage, Watson-Crick base pairing between the crRNA and the protospacer in the phage genome targets the offender for cleavage and subsequent degradation by Cas effector proteins [14, 15]. The ability of an individual bacterium to effectively resist phage infection largely depends on the number and degree of diversity of the spacers in its CRISPR-Cas system. Empirical data show a high variability of spacer numbers (from the single digits to several thousand) and genetic diversity in a typical bacterial population [16]. A central question then concerns the nature of the statistical distribution of spacers both globally, and within a typical microbial population, as well as the corresponding role of phage infections in shaping these distributions. In this paper, we use mathematical modelling supported by data to address this fundamental question for the first time.

The existing research into statistical distribution of spacers is mostly focused on the diversity of spacer types within bacterial populations. It was shown that an increase in spacer diversity generally affects population stability [10, 17] and that the effectivness of the spacers can be strongly influenced by spacer diversity [16] in single phage experiments. Recently, rank abundance curves of spacer types were constructed from a mathematical model of bacteria-phage interaction in a chemostat [18], and the model was seen to show a quick drop in the abundance of rare spacers. However, to the best of our knowledge, the quantitative aspect of spacer abundance in microbial population, *i*.*e*. the distribution of the total number of spacers in CRISPR arrays among individual bacteria, still remains largely unaddressed (but see [19] where Class I CRISPR-Cas systems were found to tend to follow a geometric distribution, although the sample size was rather small). From the previous studies, it is known that the number of spacers increases as a result of interaction with microbial viruses and decreases due to non-specific deletions during genome replication [20]. In a population of microorganisms, cells equipped with long arrays against diverse phages may be expected to increase in numbers when phages are present, due to new spacers acquired by each CRISPR. Also, the anti-selection of cells with insufficient CRISPR arrays, *i*.*e*. those lacking an appropriate spacer, usually results in an increase of the average length of CRISPR arrays in the surviving population. On the other hand, the maintenance of large CRISPR arrays may impose higher metabolic costs [20], thus reducing their replication rate [16].

One may expect that in a generic phage-microbe system the total number of spacers in CRISPR arrays should cluster around some mean value and quickly decay away from it. Here we show, however, that the statistical distribution of the number of spacers in CRISPRs in both bacteria and archaea exhibits surprising ‘fat-tail’ behaviour. In this case, the probability of observing a large number of spacers remains significant, since the number of spacers *i* is described by a power law distribution *p*(*i*) ∼ *i*^−*α*^. An important property of power law distributions is the absence of statistical moments of order *n*, where *n* > *α* − 1 (*i*.*e*. if *α* < 2 the average is infinite). Fat tail patterns in statistical distributions have been previously observed in various natural and artificial complex systems where rare catastrophic events cannot be discounted as negligible, including financial markets, animal movement, earthquakes, and terrorist attacks [21].

Our study combines a thorough statistical analysis of metagenome data and the implementation of a mathematical model which fits the empirical data. The mechanism of the emergence of the power law distribution predicted by the model is similar to that of the ‘rich-get-richer’ paradigm reported in other systems. The model also suggests that a trade-off between the CRISPR array length and the reproductive success of the cell prevents the proliferation of all-resistant super microbes. We argue that this may explain the successful infection of bacteria and archaea by phages, despite the presence and effectiveness of CRISPR-Cas immune systems.

## Materials and methods

### Model equations

We construct a mathematical model where a stable distribution of the spacers in CRISPR arrays emerges as a result of interaction between bacteria and phages. Our model takes into account the key mechanisms of the molecular processes of spacer acquisition and loss as well as host death and replication [2, 3, 13–17, 20, 22–26]. Note that for some bacteria possessing spacers, their CRISPR machinery inside the cell can be non-active (e.g. orphan arrays, loss of cas genes, decay in CRISPR repeats), so the cell will not able to acquire and actively uptake novel spacers. [27] In our model, we always assume that all bacteria with CRISPR can actively use this machinery.

**Fig 1:**
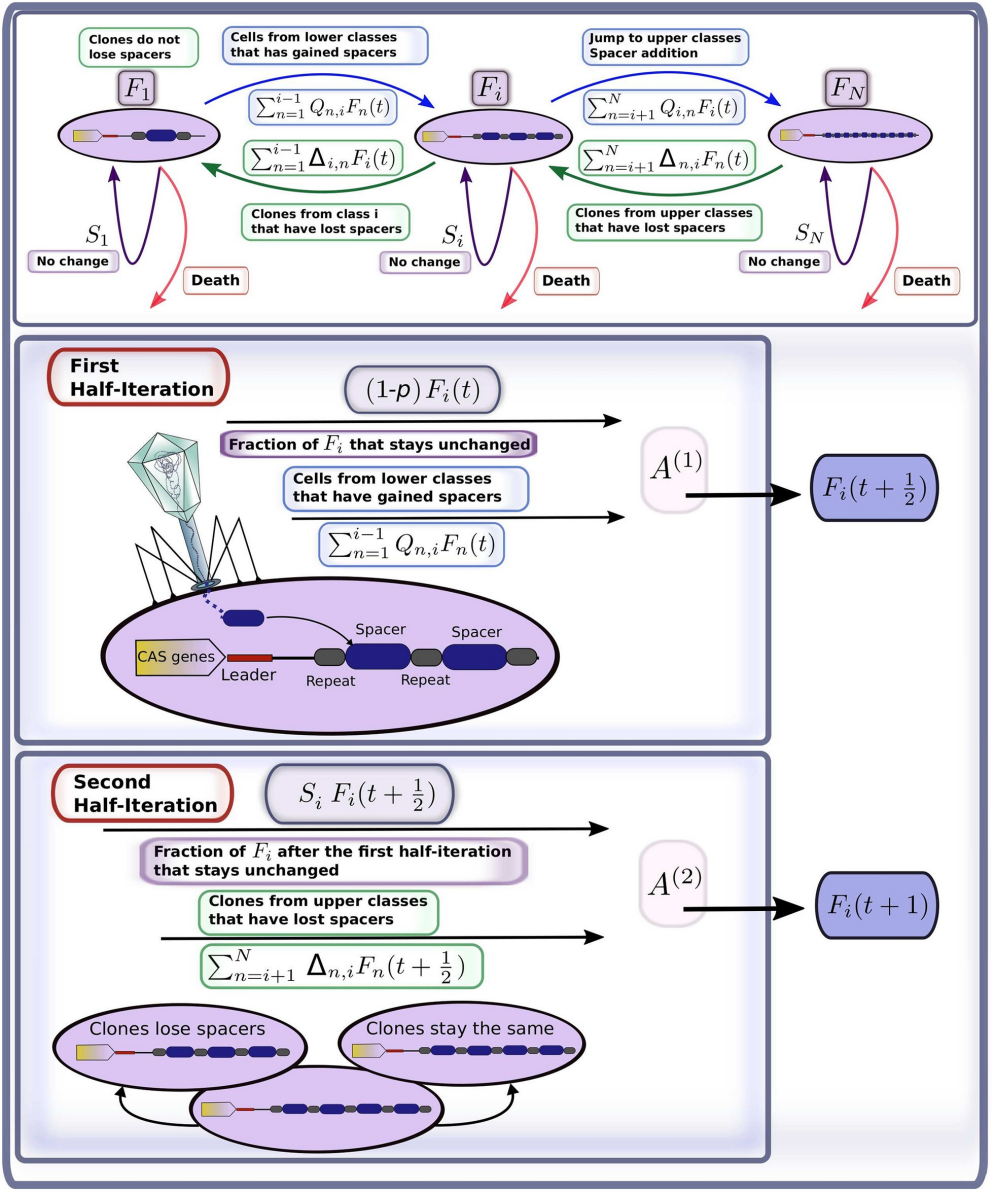
Simplified flowchart of the mathematical model: Adjustment of class size *i* at time iteration *t*. The first half-iteration describes the infection in the case the cell meets a phage (with probability *p*). Possible outcomes of this stage are: gaining *j* − *i* spacers (with probability *Q*_*i*,*j*_), death of the cell, and survival without gaining any spacers (with probability 1 − *p*). The second half-iteration mimics the replication stage. Replication can result in loss of *i* − *j* spacers in the daughter cell. The parent cell is assumed to always keep the same number of spacers. The loss or conservation of spacers number at this stage are described, respectively, by Δ_*j*,*i*_ and *S*_*i*_. Details on the parameterization of *Q*_*i*,*j*_, Δ_*j*,*i*_, and *S*_*i*_ are provided in the supplementary material.

For simplicity, the model assumes that each microbial genome contains a single CRISPR array. Since our main focus is on the statistical distribution of the number of spacers present in the CRISPR array, we do not distinguish here between different types of spacers or their origin. We use a discrete time modelling approach based on Markov chains, following the model flowchart provided in Fig 1. A detailed description of the model is given in the supplementary material. We assume that the maximal number of spacers that the bacteria may have cannot exceed some fixed large number *N*. We group all individual cells into classes corresponding to the total number of spacers present in their CRISPR array, *i* ∈ {1, …, *N*}. The abundance of class *i* at time *t* is described by *F*_*i*_(*t*). Numbers *F*_*i*_(*t*) compose the state vector **F**(*t*) = 〈*F*_1_(*t*), …, *F*_*N*_(*t*)〉. We assume the total population size is settled on some constant environmental carrying capacity, *i*.*e*. Σ_*i*_ *F*_*i*_(*t*) = *F*_*const*_.

Each discrete time step *t* is split up into two stages (Fig 1). The first stage is related to infection by the phage, which can result in either failure or success of the immune defence; signifying either death of the cell or its acquisition of spacers, respectively. The given stage is modelled by:

$$\begin{array}{c} F\left(t+\frac{1}{2}\right)=F\left(t\right)A^{\left(1\right)}, \end{array}$$ (1)

where non-zero elements of the transition matrix **A**^(1)^ describe the probability of acquisition of *j* − *i* spacers by cells from class *i* (terms *Q*_*i*,*j*_ > 0, *j* > *i*), the probability that cells from each class *i* die or the probability that the cell stays in the same class *i* (terms 1 − *p*). The latter case accounts for microbes that simply did not encounter a virus at the considered time step.

The second stage consists of the replication of the surviving cells, continuing until the carrying capacity is reached and the death of those cells whose immune systems failed to resist infection is compensated for. We assume that during replication the parent cell—that inheriting the leading DNA strand—does not lose any spacers. On the contrary, the daughter cell, inheriting the lagging strand of the parent genome, may lose spacers. This is described by the following equation

$$\begin{array}{c} F\left(t+1\right)=F\left(t+\frac{1}{2}\right)A^{\left(2\right)}=F\left(t\right)A^{\left(1\right)}A^{\left(2\right)}, \end{array}$$ (2)

where the matrix **A**^(2)^ is constructed of elements Δ_*j*,*i*_ > 0 describing the transition from upper to lower classes (*i* < *j*) and the diagonal elements *S*_*i*_ (*i* = *j*) giving the fraction of class *i* of cells that keep the same number of spacers including the parent cells.

The elements of the transition matrices **A**^(1)^ and **A**^(2)^ depend on the number of spacers *i* as well as model parameters. In the supplementary material, we show that under the model assumptions, *Q*_*i*,*j*_, Δ_*j*,*i*_, and *S*_*i*_ can be parameterised as follows

$$\begin{array}{c} \begin{array}{c} Q_{i,j}=p[q_{i}\{hP\left(\mu_{n},j-i\right)+\left(1-h\right)gP\left(\mu_{m},j-i\right))\}\\ \;\;+\left(1-q_{i}\right)sP\left(\mu_{k},j-i\right)], \end{array} \end{array}$$ (3)

$$\begin{array}{c} \Delta_{i,j}=N_{\nu }\nu_{i}P\left(\mu_{\delta }\left(i\right),i-j\right), \end{array}$$ (4)

$$\begin{array}{c} S_{i}=1+N_{\nu }\nu_{i}P\left(\mu_{\delta }\left(i\right),0\right), \end{array}$$ (5)

where the constant parameters are: *p*, the probability to encounter a phage; *h*, the probability that the protospacer exactly matches a spacer in the CRISPR array and the spacer works perfectly; this parameter also describes the scenario of infection by a non-functional virus. The parameter *s* denotes the probability to survive a viral infection in the absence of spacers (for example, this can be due to infection by non-functional virus inactivated by UV radiation, working like an antidote [28, 29]); *g* is the probability that an inefficient spacer (*e*.*g*. effector proteins bind with a lower affinity; the protospacer or PAM has mutated, the spacer is located at the distal end of the CRISPR array, *etc*) does not cause the death of the cell. The parameters depending on the number of spacers are as follows: *ν*_*i*_ are the replication rates of the bacteria, which are scaled by the factor *N*_*ν*_ to compensate for the number of deaths at each iteration (see supplementary material); *q*_*i*_ represents the probability that the CRISPR library contains a spacer exactly or inexactly matching the protospacer from the genome of the given virus strain infecting the cell. Note that *q*_*i*_, along with *h* and *g*, implicitly takes into account biodiversity of spacers since we assume that it is an increasing function of the array length *i*. The probabilities *P*(*μ*_*k*_, *i* − *j*), *P*(*μ*_*m*_, *i* − *j*), and *P*(*μ*_*n*_, *i* − *j*) describe spacer addition in the following three cases: (a) no spacer is present (index *k*), (b) spacers are present, but can work inefficiently (index *m*), or (c) the required spacer is present and it works perfectly (index *n*). The probability *P*(*μ*_*δ*_(*j*), *j* − *i*) denotes deletion of *j* − *i* > 0 spacers.

Our model does not differentiate spacers in regard to their position in the CRISPR array. The assumption is supported by the work by McGinn and Marraffini [30] which shows that spacers located downstream in the CRISPR array can still provide strong immunity when they constitute some critical proportion of the culture infected (in this study we assume that this is always the case).

**Fig 2:**
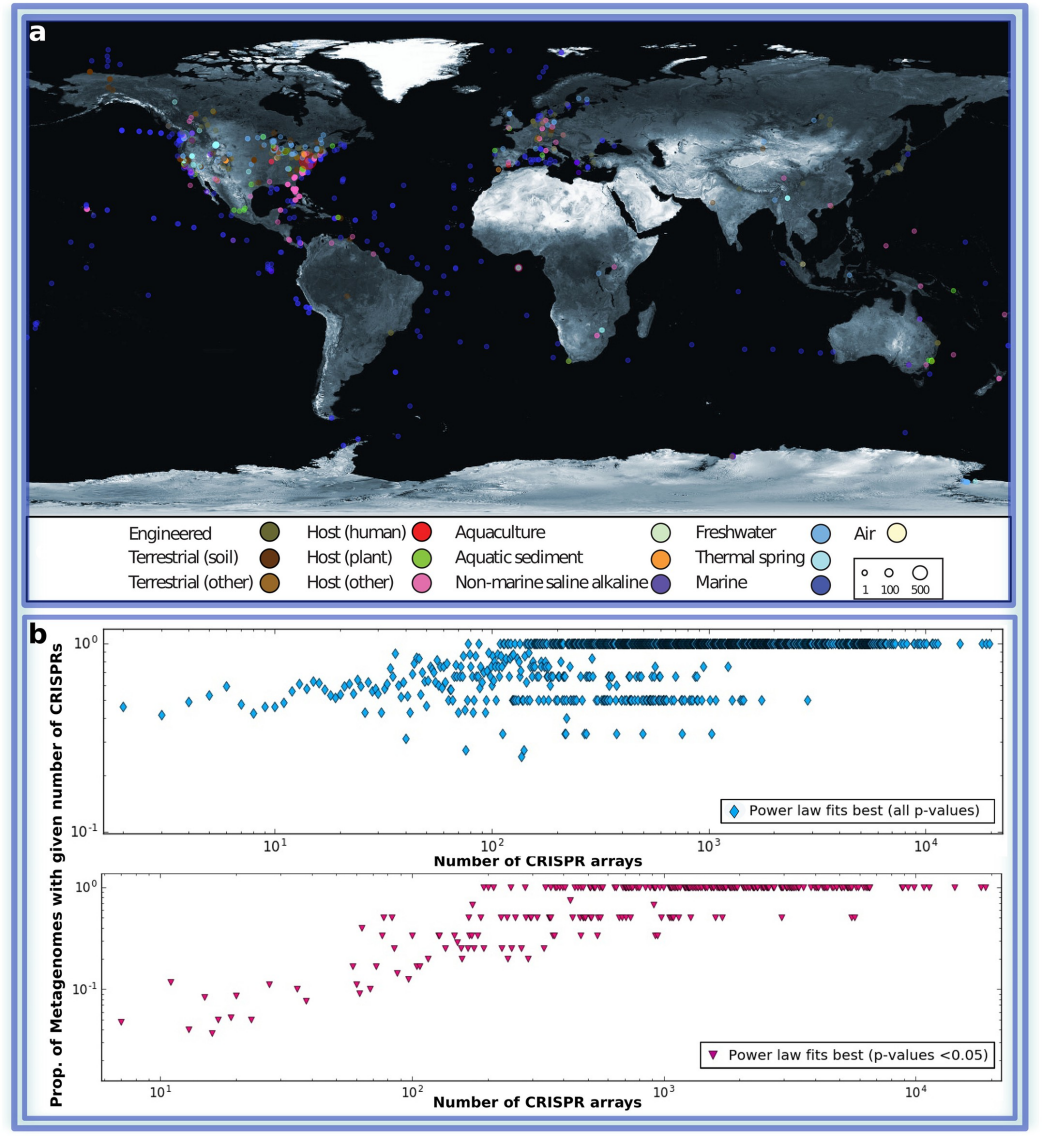
(**a**) World map displaying 3,585 out of 3,858 public metagenomic datasets, for which geographic location is reported, and classified into 13 distinct habitat types (see the color code in the caption) from which we extracted the CRISPR spacer information provided by IMG/M. The total number of samples from a single location is shown with different circle sizes, as indicated in the inset box. For more detail see Table 1 The World map taken from https://visibleearth.nasa.gov/images/57752. (**b**) The presence of power law distributions of spacers within CRISPR arrays of individual metagenomes. Each point represents all the metagenomes containing a given number of CRISPR arrays. The ordinate value shows the proportion of metagenomes for which either a truncated power law or a non-truncated power law was the best fit.

We made the following assumptions. The number of spacers added during one iteration event is assumed to be exponentially distributed, described by *P*(*μ*_*k*_, *i* − *j*), *P*(*μ*_*m*_, *i* − *j*), or *P*(*μ*_*n*_, *i* − *j*) with mean values given by *μ*_*k*_, *μ*_*m*_, and *μ*_*n*_, respectively. The use of exponential law to describe adding of spacers has some empirical support in the literature, for example, in the study by Li and co-authors [31] the band intensity in the agarose gels is a monotonic function of the number of DNA copies, it seems to behave in a similar way as the exponential distribution, at least qualitatively (see Fig 2B in the cited paper).

Microbial viruses have very high mutation rates and a single mutation in the protospacer or the protospacer adjacent motif (PAM) in the phage genome may allow the virus to escape detection [13, 26]. However, inefficient spacers containing several mismatches might prime new spacer acquisition more efficiently, compared to naive adaptation [32]. In the case of the perfect match of spacer and the phage protospacer we expect successful priming to occur (in this case often referred to as Interference-driven spacer acquisition); this has empirical evidence [25, 33]. In this light, we assume that *μ*_*n*_ ≈ *μ*_*m*_ ≫ *μ*_*k*_. New spacers are recruited from different regions of the phage genome [16], so that a phage overcoming resistance of a host with an initiated CRISPR array is very unlikely. During replication events we assume that the cell inheriting the leading DNA strand does not lose any spacers. Daughter cells, inheriting the lagging strand of the parent genome, lose spacers according to an exponential distribution with mean value *μ*_*δ*_(*i*) = *S*_*L*_*i*, where *S*_*L*_ is the fraction of existing spacers that will be lost. The loss of spacers is modelled *via* an exponential distribution which has partial support in previous studies. For example, the study by Kupczok and Bollback [34] combining a theoretical model and empirical data shows that the maximum of the probability density function (known as the mode of the distribution) of the loss of spacers is observed for either 0 and 1 spacers. Larger numbers of lost spacers are also possible, but at a smaller probability (see also some empirical demonstration of the possibility of loss of multiple spacers [35]). The exponential distribution qualitatively describes this behaviour and we use it here for mathematical convenience.

The parameter values will generally vary across biomes and ecosystems, depending on the abundance and diversity of viruses, mutation and replication rates, *etc*. We investigated the dependence of the equilibrium probability distribution of the number of spacers *i* present in CRISPR on the values of these parameters (for mathematical details see the supplementary information). To compare our model with the empirical distributions more appropriately, we address the case of bacterial and archaeal genomes where only a single CRISPR array is present.

Finally, we should highlight that the above mathematical model can also describe a more complicated scenario of the abortive infection mechanism of CRISPR. According to this mechanism, even for the perfect match of spacer, the phage would kill the infected bacteria but will get a reduced burst size [27, 36]. In this case, instead of a single cell, our model will consider a group of neighbouring cells as a combined entity. For such cells the probability to survive the infection will be higher and will be determined by an averaged over space (volume) parameters. In this case, the spatial structure of the microbial community and spatial infection patterns will be taken implicitly. The scale-invariance of the distribution of spacers can justify this interpretations: the entire system behaves similarly to the subsystems even taken at the microlevel.

### Metagenomic samples used and collection of spacers

We used all publicly available (3,858) metagenomic datasets from the IMG/M system [37]. We manually classified all samples into 13 distinct habitat types (Table 1) based on the associated metadata, following the criteria described in IMG/VR [38]. 93% of the datasets contained a geographic location (longitude and latitude) which is visualized in Fig 2A. Visualization was done using the Processing programming language (https://processing.org) and a freely available equirectangular projection of the world map (http://eoimages.gsfc.nasa.gov) was used as a background image. Sample points are positioned by latitude and longitude coordinates of Biosamples obtained from GOLD [39].

**Table 1:** Metagenomic datasets classified by habitat types.

Metagenome Environment	Number of Samples
Air	21
Aquaculture	2
Aquatic Sediment	82
Engineered	344
Fresh Water	599
Host (human)	503
Host (other)	289
Host (plant)	275
Marine	747
Non-Marine Saline and Alkaline	152
Terrestrial (soil)	638
Terrestrial (other)	56
Thermal Springs	152
Total	3,858

We used the total number of spacers *per* contig (CRISPR-Cas array) and *per* sample generated by the IMG/M system, that uses a modified version of the CRISPR Recognition Tool (CRT) [40] algorithm described in [41].

### Complete prokaryotic genomes

**Fig 3:**
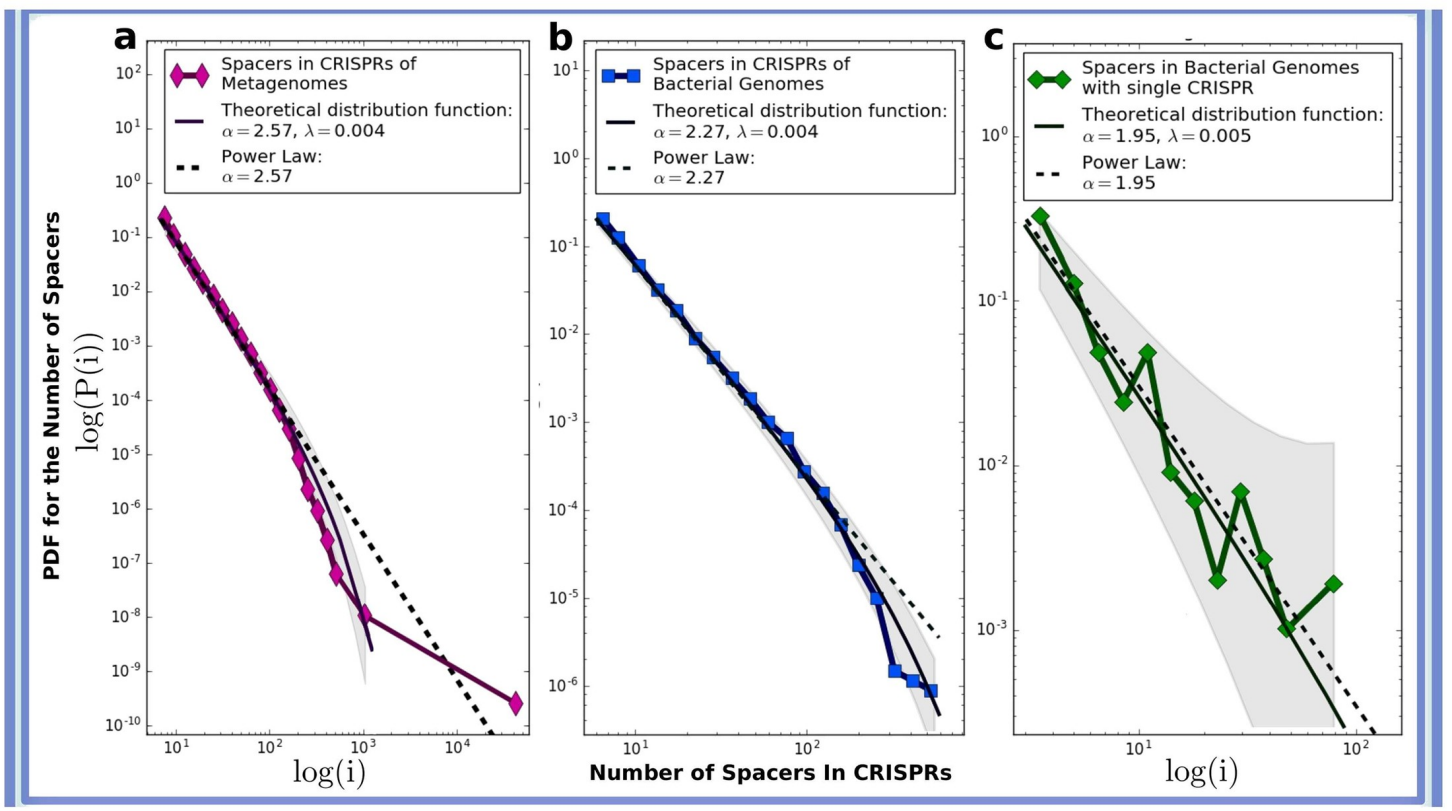
Spacer distributions in empirical genome sequences generally exhibit power law behaviour. Statistical distribution of the number of spacers across CRISPR arrays in the data combined from (**a**) 3,858 metagenomes, (**b**) 6,214 complete archaeal and bacterial genomes, (**c**) 237 complete genomes possessing only a single CRISPR array. The curves are fitted using the power law function with an exponential decay *p*(*i*) ∼ *i*^−*α*^ *e*^−λ*i*^ and are shown by solid lines. The dashed line shows the slope of the power law function. The shaded area shows 95% confidence bands.

All available bacterial and archaeal (6,214) genomes were downloaded from GenBank [42]. We used the CRISPR Recognition Tool (CRT) [40] with default settings to detect CRISPR arrays. As in the case of metagenomes Fig 3B shows the number of CRISPR arrays (y axis) possessing given number of spacers (x axis) regardless of the number of CRISPR-Cas systems in genomes. Fig 3C shows the same data but only for genomes equipped with single CRISPR array.

A better approach to the distribution of spacers would take into account the distribution of the occurrence of CRISPR systems *per* genome. This can be done for complete genomes. We should say, however, that the available database of completely sequenced prokaryotic genomes in GenBank seems to be heavily biased towards a limited number of medically important and model organisms [43]. Adjusting the effects of this bias from the empirical data is a non-trivial task which itself can be subjective. Our metagenome dataset seems less biased (*e*.*g*. see Table 1). In this case, however, extracting complete genomes would largely reduce the number of points. Nevertheless, distributions in the three cases belong to the same family and are qualitatively identical (see the Results section).

### Statistical analysis

Data on the number of spacers in CRISPR arrays in metagenomes, as well as in complete genomes, were handled with Python. We used the Python package **powerlaw** [44] for analysis of fat tailed distributions in order to adequately fit the patterns observed in natural CRISPR arrays and our numerical simulations to appropriate probability density functions. To generate the results in Figs 3 and 2B, we used the discrete powerlaw.fit object. The goodness of the truncated_powerlaw fit was compared with other candidate heavy tailed distributions using distribution_compare, which calculates Loglikelihood ratio for two given distributions *p*_1_ and *p*_2_ as $R=\sum_{i=1}^{n}(\text{ln}\;\left[p_{1}\left(x_{i}\right)-p_{2}\left(x_{i}\right)\right])$. *P*-value is calculated as $p=\frac{1}{\sqrt{2\pi n\sigma^{2}}}\left(\int_{-\infty }^{-|R|}e^{\frac{-t^{2}}{2n\sigma^{2}}}dt+\int_{\left|R\right|}^{+\infty }e^{\frac{-t^{2}}{2n\sigma^{2}}}dt\right)=\frac{2}{\sqrt{\pi }}\int_{\frac{R}{\sigma \sqrt{2n}}}^{+\infty }e^{-t^{2}}dt$, where $\sigma^{2}=\frac{1}{n}\sum_{i=1}^{n}(\left[p_{1}\left(x_{i}\right)-p_{2}\left(x_{i}\right)\right]-[\frac{1}{n}\sum_{i=1}^{n}\text{ln}\;p_{1}\left(x_{i}\right)-\frac{1}{n}\sum_{i=1}^{n}\text{ln}\;p_{2}\left(x_{i}\right)])$ [21, 44]. Loglikelihood ratios (positive if the data is more likely in the truncated_powerlaw distribution) and significance *p*-values are listed in Table 2. If *p* is small (common practice is to consider *p* < 0.05) then the value for R is unlikely to be a chance result and its sign can be trusted as an indicator of which distribution fits the data better. Pure power law distribution is a subcase of truncated power law, whereas exponential distribution is a subcase of stretched exponential. Thus the method has to be correct for comparisons of both nested and non-nested distributions. Loglikelihood ratio test is shown to be correct for both cases for the distributions we considered [45].

**Table 2:** Comparison of the truncated power law *p*(*x*) ∼*x*^−*α*^ *e*^−λ*x*^ to other heavy-tailed candidate distributions in fitting empirical distribution of spacers. Positive Loglikelihood ratios indicate that the Truncated Power Law fits the empirical data better than the candidate distribution function.

	Metagenomes		Complete Genomes	
	Loglikelihood ratio	*P*-value	Loglikelihood ratio	*P*-value
Exponential $p\left(x\right)=\frac{1}{\lambda }e^{x/\lambda }$	62981.65	0.0	3877.26	4.90x10^−159^
Stretched Exponential $p\left(x\right)∼e^{-\lambda x^{-\beta }}$	1306.33	8.23x10^−3^	35.15	1.17x10^−16^
Longnormal, *μ* > 0 $p\left(x\right)=\frac{1}{x\sigma \sqrt{2\pi }}e^{-\frac{{\left(\ln x-\mu \right)}^{2}}{2\sigma^{2}}}$	2909.08	1.47x10^−56^	150.64	7.82x10^−28^
Power Law *p*(*x*)∼*x*^−*α*^	536.83	0.0	58.05	0.0

### Mathematical model and simulations

**Fig 4:**
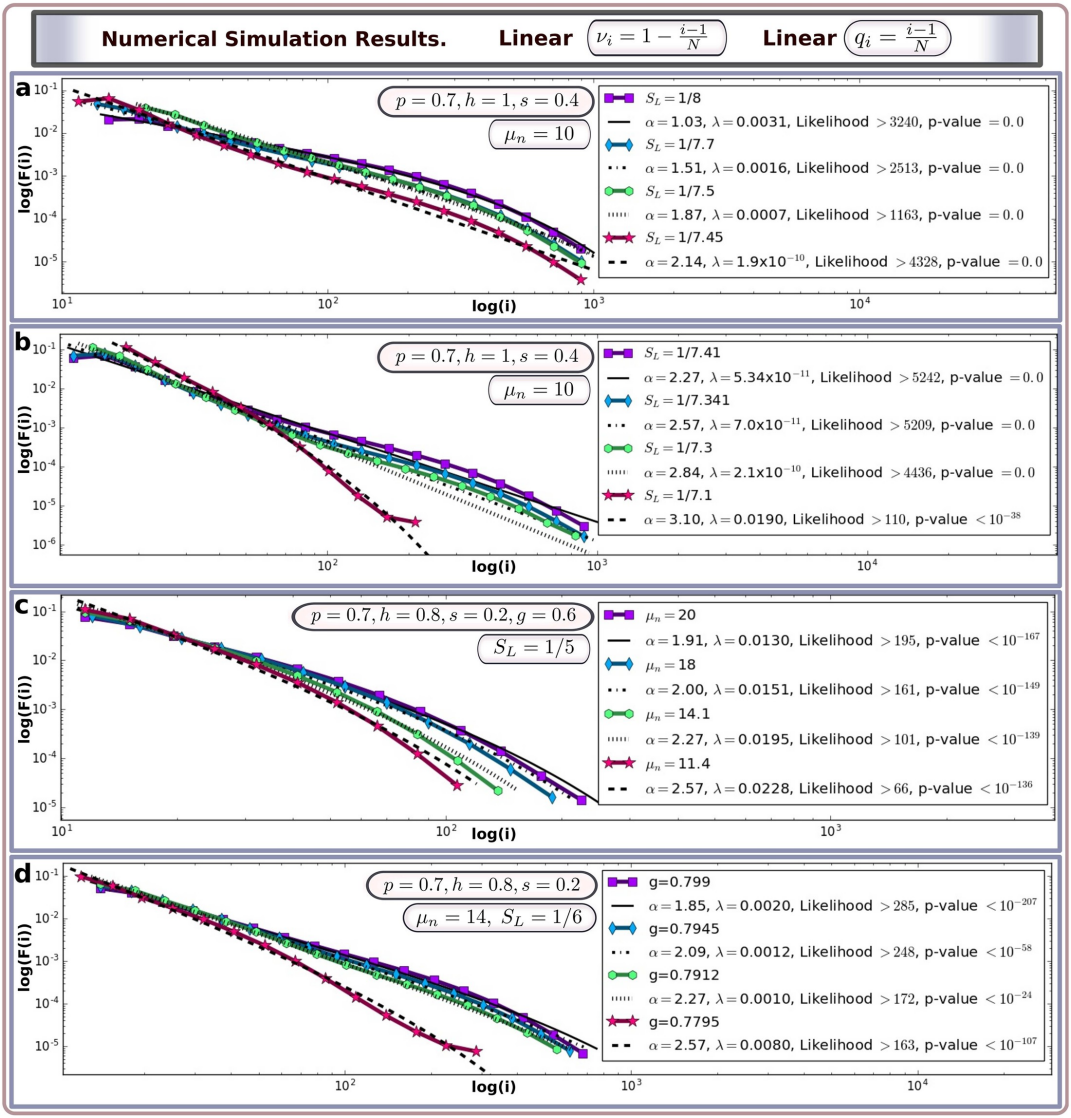
Statistical distribution of spacers in CRISPR array predicted by the mathematical model with linear parameterisations of *q*_*i*_ and *ν*_*i*_. In all graphs, colored lines with dots represent the final distribution F* in the model. The thin, dashed, dotted, and dash-dotted lines show the fitting of the power law distribution with exponential cutoff given by *p*(*i*) ∼ *i*^−*α*^ *e*^−λ*i*^. In the labels, likelyhood stands for Loglikelyhood ratio of truncated power law distribution to the second best fit. (**a,b**) For the fixed parameter values *p* = 0.7, *h* = 1, *s* = 0.4 and *μ*_*n*_ = 10, increasing the fraction of spacers lost during replication from *S*_*L*_ = 1/8 to *S*_*L*_ = 1/7.1 increases the power law exponent from *α* from 1.03 to 3.1. (**c**) For the fixed parameter values *p* = 0.7, *h* = 0.8, *s* = 0.2, *g* = 0.6 and *μ*_*n*_ = 10, decreasing the average number of spacers gained due to viral infection *μ*_*n*_ from 20 to 11.4 increases the power law exponent from 1.91 to 2.57. (**d**) For the fixed parameter values *p* = 0.7, *h* = 0.8, *s* = 0.2, *S*_*L*_ = 1/6 and *μ*_*n*_ = 14, decreasing the probability of survival in the case of a mutated protospacer from 0.799 to 0.7795 increases *α* from 1.85 to 2.57. In all of the above cases we assume that 100*μ*_*k*_ = *μ*_*m*_ = *μ*_*n*_. Decreasing the value of *μ*_*m*_ has a similar effect as decreasing *μ*_*n*_ (see Fig F in S1 Text).

Detailed derivation of the expressions for the components of the transition matrices in **A**^(1,2)^ is provided in the supplementary material. The iterative numerical simulation was implemented in MATLAB. The maximal number of spacers was considered to be *N* = 1, 000, the total population size was set as *F*_*const*_ = 10^6^. At each step, the replication rate was multiplied by the scaling factor *N*_*ν*_ to preserve the constant population density at *F*_*const*_; the value of *N*_*ν*_ is computed using the corresponding formula in the supplementary material. To find the stationary distribution F* we both used direct iterations (after roughly *t* = 4000 the distribution becomes approximately stationary) and solved the matrix equation F* = F*A = F*A^(1)^ **A**^(2)^. Note that the actual value of *F*_*const*_ does not affect the modelling results: its re-scaling by the factor of 10, 100, *etc*, does not modify the final distribution of spacers if the simulation time is large enough. Moreover, the statistical distribution (measured as percentage of spacers) would remain the same even in the absence of the upper bound for *F*_*const*_. Fitting of curves to stationary distributions obtained in the model was done using the Python package **powerlaw** [44]. The folder S1 Matlab Code contains a MATLAB code which produces the statistical distributions of spacers corresponding to Fig 4 in the Results section.

## Results

### Revealing statistical distribution of spacers from data

We conducted extensive empirical data analysis of the statistical distribution of the number of spacers across CRISPR arrays in 3,858 metagenomes [37] from various geographical locations and ecosystem types (2,189,103 CRISPRs and 11,724,296 spacers in total). The results are presented in (Fig 3A). Fitting the data points with standard curves shows that the distribution follows a power law distribution with exponential decay given by *p*(*i*) ∼ *i*^−*α*^ *e*^−λ*i*^, with *α* = 2.57 and λ = 0.004 ±0.0013. Our estimate for uncertainty of the power law exponent provide the 95% confidence interval to be 2.47 < *α* < 2.67.

In order to exclude the influence of possible artefacts of sequencing or assembling technique for metagenomic reads, which may corrupt the resulting pattern, we also analyzed the distribution of spacers across CRISPR arrays in all complete archaeal and bacterial genomes available in GenBank (6,214 genomes, 101,471 CRISPRs, 439,829 spacers) [42]. Our results are shown in Fig 3B. We found that the distribution can be approximated by a power law with exponential cutoff for the parameters *α* = 2.27, and λ = 0.004 ± 0.0014 (the 95% confidence interval for *α* is 2.20 < *α* < 2.34). We also plot the distribution of spacers for genomes where only a single CRISPR array is available (237 genomes, 1,094 spacers) [42]. We found that for bacteria and archaea, the power exponent is *α* = 1.95 ± 0.31 and the exponential decay factor is λ = 0.005 ± 0.0027 (Fig 3C). The apparent disparity with the results obtained for complete genomes with various numbers of CRISPR arrays and metagenomic data as well as a higher confidence interval for *α* in Fig 3C is likely to be explained by the small sample size.

We examined the goodness-of-fit for the distributions of the exponential, lognormal, stretched exponential and power law type (all frequently used in the literature [44]) and found that the power law distribution with an exponential cutoff fits our data better than the other distribution functions considered, with *p*-values well below 0.05 (Table 2). The exponential cutoff in the observed power law distribution occurs due to the natural limitations to the number of spacers a CRISPR array may accumulate. Details on the statistical analysis and sample collection are provided in Materials and Methods.

The power law behaviour and scale-invariance in the distribution of spacers in CRISPR can also be seen directly when CRISPR arrays in individual metagenomes are compared to data for the entire planet (Fig 2B). Individual metagenomes typically contain some number of CRISPRs and distribution of spacers *per* array can be revealed even within single metagenome. We find that in more than two-thirds of the individual metagenomes, the power law gives a better fit than other heavy-tailed distributions. However, our analysis shows that the corresponding *p*-value was < 0.05 in each comparison for only 7.75% of the datasets. This can be explained as a result of the generally low signal-to-noise ratio when there are too few CRISPRs and spacers within a metagenome.

Finally, we show (Fig 2A) the geographic location of the majority of the available metagenomic datasets (93%). The samples are classified according to the type of the habitats (13 types overall), with the distribution of the metagenomic datasets across the habitat types shown in Table 1. Thus our results regarding distributions of spacers hold for microbial communities regardless of their geographic locations and/or habitat types (see also Table A in S1 Text). As soon as the number of CRISPR arrays in the sample is sufficient for statistical inference, truncated power law distribution is clearly observed, even within a single metagenomic sample. Interestingly, combining data for several metagenomes into a single data set results in the same pattern which can be explained by the fractal nature of the considered distribution type.

### Spacer distribution predicted by the model

Using the introduced model, we run iterations for large times *t* until the distribution of spacers becomes stationary. The eventual stationarity of **F** in the model is guaranteed since the combined transition matrix **A** = **A**^(1)^ **A**^(2)^ is aperiodic and irreducible by construction. The stationary distribution F* is given by the principle eigenvector of **A** corresponding to the maximal eigenvalue of 1. Here we investigate the possible distributions F* which emerge in various scenarios for the dependence of the replication rate *ν*_*i*_ and the spacer matching probability *q*_*i*_ on the array size *i*.

We first consider constant values *q*_*i*_ = *q* and *ν*_*i*_ = 1, *i.e*. the replication rates and the effectiveness of the immune system do not depend of the size of the CRISPR library. We find that the resultant distribution F* is normal or skew normal in agreement with the central limit theorem. We also observe that the average number of spacers in the array increases with an increase of the average spacer gain *μ*_*n*_ and with a decrease of the fraction of existing spacers lost during replication *S*_*L*_. The corresponding graphs are shown in Fig D in S1 Text). Including the dependence of *q*_*i*_ on *i* (we consider a monotonically increasing function *q*_*i*_) results in a right-centered distribution similar to a normal distribution (supplementary material Fig E(i) in S1 Text). On the other hand, by keeping *q*_*i*_ constant and assuming the replication rate to be a decreasing function of *i* (*e*.*g*. a linear function), we obtain a left-skewed distribution F*; however, the distribution function still rapidly converges and does not exhibit fat-tailed behavior (Figs E(ii), E(iii) and E(iv) in S1 Text).

We find that in order to obtain a power law-like distribution of spacers in the CRISPR array one needs to assume dependence on *i* in both *q*_*i*_ and *ν*_*i*_. For example, by considering the simplest (linear) parameterisations *q*_*i*_ = (*i* − 1)/*N* and *ν*_*i*_ = (*N* + 1 − *i*)/*N* we observe power law distributions with an exponential cutoff, as shown in Fig 4A and 4B. This observation can be quantitatively verified by fitting the curves to the data points predicted by the model (the estimated exponents *α*, and λ are shown in the figure). We also use statistical analysis to compare some other candidates for heavy-tailed distributions using the maximum likelihood principle [44]. We find that a power law with an exponential cutoff is the best fit in the case where a left-centered heavy-tailed distribution emerges and persists under small variation of the parameters. The fat tailed behaviour is not strongly dependent upon the concrete parameterisations of *q*_*i*_ and *ν*_*i*_: the pattern persists for some monotonically increasing functions *q*_*i*_ (*e*.*g*. logistic) and monotonically decreasing functions *ν*_*i*_ (*e*.*g*., Gaussian centered at zero). The assumptions of monotonicity in the dependence of *q*_*i*_ and *ν*_*i*_ are natural since the possession of a large CRISPR array may incur costs for the cell, and a longer CRISPR library would normally signify a larger biodiversity of spacers, signifying a higher probability of finding an appropriate spacer [16, 20, 46].

Finally, we investigated the dependence of the spacer distribution exponents, *α* and λ on key model parameters. Overall, we found that a truncated power law distribution can be observed in the model within a large range of model parameters. An increase in the fraction of spacers lost during replication *S*_*L*_, was seen to generally lead to a gradual increase in the power law exponent *α* and a decrease in the maximum number of spacers in the population *i*_*max*_ (*i*.*e*. the number of non-empty classes *F*_*i*_, see Fig 4A and 4B). An increase in the average number of spacers gained *μ*_*n*_ generally leads to smaller power law exponents *α* (Fig 4C), and the same dependence is found for variation of the parameters *μ*_*m*_ and *μ*_*k*_ (Fig K in S1 Text). A reduction in *g*, the probability that an inefficient spacer does not cause the death of the cell, results in an increase in *α* (Fig 4D). We should note that variation in the model parameters (*S*_*L*_, *μ*_*n*_, *μ*_*m*_, *μ*_*k*_, *h*, *g*) can result in either gradual or abrupt, bifurcation-like changes in the slope of the spacer distribution. Corresponding examples are provided in the supplementary material (Figs F-K in S1 Text). Finally, by varying key parameters, the model is able to reproduce the CRISPR spacer distributions shown in Fig 3 for metagenomes (*α* = 2.57), all bacterial genomes (*α* = 2.27), and bacterial genomes with a single CRISPR array only (*α* = 1.95). The corresponding graphs are provided in the supplementary material.

## Discussion

Power law distributions characterised by heavy or fat tails are observed in a wide range of natural phenomena and complex systems from earthquake magnitudes [47] and energy cascades in turbulent eddies [48] to individual net wealth [49], severity of terrorist attacks [50], and market crashes [51]. Power law behaviour is also a well-known feature in biological systems, and determining the mechanisms of its emergence and maintenance is critical for understanding connections between different levels of biological organization in biochemistry, physiology, epidemiology (*e*.*g*. spread of infectious diseases including the current COVID-19 case [52]) and ecology, and it has important consequences for biological evolution [53–57]. In this paper, we use a combined empirical and theoretical approach to reveal for the first time that patterns of power law behaviour can be seen in CRISPR-Cas systems, which represent the main immune defence mechanism for about half of all bacterial species and in about 90% of archaea [1].

We investigated the number of spacers in CRISPR arrays across global metagenomes and demonstrated that they follow a power law distribution with an exponential cutoff (Figs 3 and 2). Our estimates of the power law exponents showed close values for various ecosystems (natural, artificially engineered, or in humans), varying between *α* = 2 and *α* = 3, with only small difference between geographic locations and climates (see supplementary material). Interestingly, for some biological systems such as terrestrial non-soil ecosystems, the power law exponent is estimated to be below 2, indicating that most cells are assigned to the tail of the distribution in terms of their spacers (see supplementary material). Mathematically speaking, for such distributions the average CRISPR size should be infinitely large, although in reality, due to the exponential cut-off the average size is large but finite. Overall, our findings indicate a very slow decay of the numbers of spacers in distributions and the existence of a non-negligible amount of microbes with a very large amount of spacers: the average number of spacers in a CRISPR array was seen to be 12.73, with a standard deviation of 14.15. In our metagenome data the maximum number of spacers found in a single CRISPR array is 1,131. Given estimates of the number of prokaryotic cells on the Earth as being ∼ 10^30^ [58, 59], and assuming a probability distribution function *p*(*i*) = *i*^−2.57^ *e*^−0.004*i*^, we can extrapolate that the longest possible CRISPR on the Globe should contain approximately 11,300 spacers.

We found that the number of spacers in the distribution quickly decays when the array length reaches several hundreds. This corresponds to more than 20,000 base pairs (bp), whereas the longest prokaryotic protein coding gene does not exceed 6,000 bp [60]. The longest prokaryotic transcript deposited in GenBank is 18,651 nucleotides and belongs to high GC content species [61]. Among other factors, insufficient processivity of RNA polymerase may explain the observed drop in the number of spacers in the distribution. On the other hand, one may expect a high processivity of RNA polymerase when the microbial genome contains longer CRISPR arrays. This important observation should be taken for further studies and used for applications, for example in biotechnology, since the high efficiency transcription machinery is expected to be present in organisms which can afford high lengths of CRISPR arrays.

Empirical distributions of spacers can be represented by the generic mathematical model considered here (assuming that in all cells containing spacers, the CRISPR machinery is always active). According to the model, the emergence of heavy-tailed behaviour is the outcome of interaction between (i) the positive feedback between the number of spacers and the rate of increase in the length of the CRISPR array, and (ii) the negative trade-off between the replication rates of cells and the CRISPR array length. This mechanism has a clear biological rationale: cells possessing a larger number of spacers will generally have a higher chance of surviving phage infection, which should result in natural selection for such cells in the face of persistent viral infection pressure [20]; however, fitness costs required to maintain large CRISPR arrays would reduce the replication rate of such cells [16, 46]. Dilution of the most useful effector Cas complexes in large CRISPR arrays has been suggested as one possible source of a fitness cost of large spacer numbers [46].

The literature on the emergence of fat tail distributions in natural and engineered systems identifies a handful of plausible scenarios [49]. The mechanism occurring in our CRISPR-Cas model is somewhat close to the classical Yule model (also known as Yule-Simon scenario), which is known as the ‘rich-get-richer’ principle [49, 62]. According to this principle, the population of cities, number of citations of research articles, personal wealth, bestseller purchases, *etc* should increase in proportion to their current numbers. In the CRISPR systems, spacers addition is facilitated in cells with larger arrays: their survival rate is higher and mathematically this is described by assuming the effectiveness of the immune system *q*_*i*_ to be an increasing function of *i*. Note that some more detailed mathematical models explicitly considering spacer diversity confirm an increase (although nonlinear) of the efficiency of CRISPR with the size of the system [63]. However, the mechanism of heavy tail formation in our model deviates from the classical Yule-Simon scenario, in some respects. Indeed, having an increasing *q*_*i*_ is not enough: this would result in having a bias towards cells with very long CRISPR arrays since only the ‘richest’ members of populations with *i* ≈ *N* will dominate (see Fig D in S1 Text). Such a situation is not observed empirically (Fig 3). Introducing replication rates *ν*_*i*_ decreasing with the number of spacers rectifies the situation and produces the observed heavy tails.

Having fat tails in the distribution of spacers in CRISPR systems has its important biological consequences which we need to know to better understand bacteria-phage interaction. In microbial populations (using CRISPR as a defence mechanism) there will be always a considerable proportion of individuals with a large number of spacers. The accumulation of spacers in the tail of the distribution is a direct consequence of the power law dependence (note that the exponential cut-off reduces spacers numbers in the tail as compared to a ‘pure’ power law distribution). As such, in the case of an occasional heavy infection by phages, bacterial cells possessing large numbers of spacers will survive, assuming that CRISPR length positively correlates with spacer diversity; these cells can then serve as an internal dynamic refuge for the whole population. Metaphorically, one might imagine such cells performing a similar role to microbial ‘stem cells’: after the disastrous phage infection, the entire population will be able to recover. Such survival of the microbial population would not be possible in the case of a Gaussian distribution of spacers with the proportion of cells with large number of spacers dropping off very quickly (Fig D in S1 Text).

On the other hand, our model predicts that an increase in the metabolic costs of replication of cells with long CRISPR arrays should reduce the growth rate of such microbes. The rate of mortality unrelated to infection in such bacteria can also be high. As a result, we can hypothesise based on our mathematical model that all-resistant super microbes (among those using CRISPR as a defence mechanism) should be very rare in nature, despite the possession of a strong immune defence. All-resistant super microbes may be more vulnerable to environmental challenges such as oil spills or climate change, *etc*. This is due to low fitness (*i*.*e*. a low replication rate) of such microbes, and in the case of such challenge microbial population may go extinct as a result of subsequent phage invasion. This may explain the successful proliferation of phages observed in the wild and in humans, despite the potential for cells to build a long CRISPR library and become super resistant. As a general principle, the addition of 4-6 extra spacers should not affect microbial fitness in terms of growth rate (Dr. Rodolphe Barrangou, personal communication). Although there is some available information on possible trade-offs between the replication rate and natural mortality of microorganisms and the size of the CRISPR system [16, 46], these remain poorly understood overall. We should also admit that CRISPR is not the only mechanism of bacterial immunity and there are several other means to mount resistance against phages, for example as phase variation, and others [27, 64].

Another consequence of the power-law in CRISPR-Cas systems is the possibility of self-criticality. Self-criticality phenomena occur when parameters of physical or biological system are tuned, for example *via* a feedback with the environment, in such a way that the statistical distribution of the considered quantity in the system exhibits a power law [49, 65]. In our model, however, we do not observe an exact phase transition phenomena as in the classical self-criticality paradigm. However we did find that the transition to spacer distributions with a pronounced power law structure generally occurs for a very small variation of model parameters (see Figs G-I in S1 Text). Empirical data show robust fat tail patterns across many different ecosystems. While our model parameters may be tuned to produce a variety of other outcomes, it appears that the dynamics of real ecosystems impose parameter constraints that produce a fat tail distribution of spacers. This may be through coupling or feedback loops among parameters that are held constant in our model.

Our model of CRISPR-Cas systems helps to better bridge two major hypotheses about the driving forces of biological evolution and speciation: the Red Queen and the Court Jester paradigms. According to the Red Queen model, the outcome of evolution primarily occurs as a result of biotic interaction and competition, for example as in host-parasite coevolution [66]. This is believed to be a relatively rapid evolutionary process. In the CRISPR system, such coevolution is likely to be limited to the less-resistant subpopulation with relatively few spacers. The resultant distribution of spacers (even taking into account possible mutations in the phage population) will be at equilibrium for a relatively long time period. According to the Court Jester paradigm, evolutionary changes such as speciation occur as a rare event in response to an unpredictable change in the physical environment [67]. For the CRISPR system, this would imply that alteration in the parameters caused by environmental change should affect the entire microbial population, most severely affecting those located in the tail of the spacer distribution. Such events may result in unpredictable mass extinction of microbes. Newly established populations may reform the same ecosystem, or they may organize into new interspecies relationships, which may create or extinguish some ecological niches, depending on various biotic and abiotic factors. Thus, our model has important implications for both the Red Queen and the Court Jester scenarios.

Finally, we would like to mention a few immediate extensions of the current study both in terms of theory and empirical work. Firstly, one can extend the current mathematical model to explicitly include the impact of the diversity of spacers on their statistical distribution (the importance of spacer diversity was emphasized in a few previous works) [16, 63]. For revealing statistical distribution of spacers from data (GenBank), it would be important to take into account the occurrence of CRISPR systems *per* genome. This will require to adjust the currently existing strong bias towards a limited number of medically important and model organisms. Future experimental work should include a more detailed quantitative description of the number of spacers lost or added at each survival of the viral attack, so we can justify the use of a particular distribution (surprisingly enough, this is currently outside the mainstream of CRISPR studies). We suggest that the correlation between the replication rate and the length of CRISPR array should be explored experimentally and revealing a strong positive correlation would support our conclusion of why supermicrobes are rare in nature. Conducting the mentioned experiments will allow us to provide an ultimate confirmation of the underlying mechanism of the emergence of fat tail.

Also, a spatially explicit model—considering the spatial heterogeneity of cells—would be essential to take into account the abortive infection scenario of CRISPR, for example building on the study by Haerter and Sneppen [68]. It was recently shown that space structuring plays an important role in CRISPR [69], therefore explicitly modelling spatial dynamics of bacteria-phage interaction should be a vital continuation of the current study.

## Conclusion

In this paper, we show that the size of CRISPR arrays (the number of spacers) in microbial populations generally follows a power law distribution with an exponential cut-off: here we apply a combined metagenomics data and mathematical modelling approach. The main biological relevance of this finding is that we can now explain the rarity of all-resistant super microbes (among those using CRISPR as a major defence mechanism): the model predicts that strong immunity of such microbes should substantially reduce their fitness (replication rates). We argue that the success of phages in nature to counterbalance an efficient immune system such as CRISPR-Cas is possible because of (i) the rarity of microbes with long spacer arrays and (ii) very low replication rates of such microbes. Important implication of our findings is that microbial communities may become extinct due to phage invasions in the case of environmental changes.

## Supporting information

S1 Text **Fig A. Flowchart of the mathematical model presenting possible outcomes of bacteria-phage interaction and replication for bacteria having** *i* **spacers. Fig B. Schematic representation of transition between size classes (containing different spacer numbers** *i* **) at time iteration** *t*. **Fig C. Temporal evolution of the transition matrix eigenvalues. Fig D. Statistical distribution of spacers in CRISPR array predicted by the model with constant** *q*_*i*_ **and** *ν*_*i*_. **Fig E. Introducing parameter dependence on the CRISPR array length** *i*. **Fig F. Spacer distribution for linear dependence of** *q*_*i*_ **and** *ν*_*i*_ **affected by variation of** *S*_*l*_ **and** *μ*_*n*_. **Fig G. Sensitivity of** *α* **with respect to variation of** *S*_*L*_ **(the fraction of spacers lost during replication) and** *s* **(the probability of survival if the microbe has no spacer for the virus). Fig H. Sensitivity of** *α* **with respect to variation of** *μ*_*n*_ **(the average number of spacers gained) and** *s* **(the probability of survival if the microbe has no spacer for the virus). Fig I. Sensitivity of** *α* **with respect to variation of** *h* < 1 **(the probability of protospacer match)**, *g* **(the probability of survival in the case of mutated protospacer) and** *s*. *s* ≤ *g* ≤ *h*. **Fig J. Variation of statistical distribution of spacers (linear** *q*_*i*_ **and** *ν*_*i*_ **) for different** *g* **(the probability of survival in the case of mutated protospacer). Fig K. Variation of statistical distribution of spacers (linear** *q*_*i*_ **and** *ν*_*i*_ **) for different parameters** *s*, *p*, *μ*_*m*_, *μ*_*k*_ **and** *h*. **Table A. Comparison of the Truncated Power Law** *p*(*x*)∼*x*^−*α*^ *e*^−λ*x*^ **to other heavy-tailed candidate distributions in fitting empirical distribution of spacers for combined metagenomes from samples collected in different environments**.
(PDF)

S1 Matlab Code The folder S1 Matlab Code.zip contains a MATLAB code which produces the statistical distributions of spacers as those shown in Fig 4. (ZIP)

## References

[1] Westra Edze R., Van Houte Stineke, Gandon Sylvain, and Whitaker Rachel. The ecology and evolution of microbial CRISPR-Cas adaptive immune systems. Phil. Trans. R. Soc. B 2019; 374:20190101. 10.1098/rstb.2019.0101 30905294 PMC6452260

[2] Marraffini LA. CRISPR-Cas immunity in prokaryotes. Nature. 2015;526(7571):55–61. 26432244 10.1038/nature15386

[3] Barrangou R, Fremaux C, Deveau H, Richards M, Boyaval P, Moineau S, et al. CRISPR provides acquired resistance against viruses in prokaryotes. Science. 2007;315(5819):1709–12. 10.1126/science.1138140 17379808

[4] Adli M. The CRISPR tool kit for genome editing and beyond. Nature communications. 2018;9(1):1911. 10.1038/s41467-018-04252-2 29765029 PMC5953931

[5] Selle K, Barrangou R. CRISPR-Based Technologies and the Future of Food Science. Journal of Food Science. 2015;80(11):R2367–R2372. 10.1111/1750-3841.13094 26444151

[6] Pursey E, Sünderhauf D, Gaze WH, Westra ER, van Houte S. CRISPR-Cas antimicrobials: Challenges and future prospects. PLOS Pathogens. 2018;14(6):1–8. 10.1371/journal.ppat.1006990 29902258 PMC6001953

[7] Noble C, Adlam B, Church GM, Esvelt KM, Nowak MA. Current CRISPR gene drive systems are likely to be highly invasive in wild populations. eLife. 2018;7:e33423. 10.7554/eLife.33423 29916367 PMC6014726

[8] Watson BNJ, Easingwood RA, Tong B, Wolf M, Salmond GPC, Staals RHJ, et al. Different genetic and morphological outcomes for phages targeted by single or multiple CRISPR-Cas spacers. Philosophical Transactions of the Royal Society B: Biological Sciences. 2019;374(1772):20180090. 10.1098/rstb.2018.0090 30905290 PMC6452268

[9] Gurney J, Pleška M, Levin BR. Why put up with immunity when there is resistance: an excursion into the population and evolutionary dynamics of restriction–modification and CRISPR-Cas. Philosophical Transactions of the Royal Society B: Biological Sciences. 2019;374(1772):20180096. 10.1098/rstb.2018.0096 30905282 PMC6452257

[10] Koonin EV, Wolf YI. Evolution of the CRISPR-Cas adaptive immunity systems in prokaryotes: models and observations on virus–host coevolution. Mol BioSyst. 2015;11:20–27. 10.1039/c4mb00438h 25238531 PMC5875448

[11] Edwards RA, McNair K, Faust K, Raes J, Dutilh BE. Computational approaches to predict bacteriophage–host relationships. FEMS Microbiology Reviews. 2015;40(2):258–272. 10.1093/femsre/fuv048 26657537 PMC5831537

[12] Sieber M, Gudelj I. Do-or-die life cycles and diverse post-infection resistance mechanisms limit the evolution of parasite host ranges. Ecology letters. 2014;17(4):491–498. 10.1111/ele.12249 24495077

[13] Mojica FJM, Díez-Villaseñor C, García-Martínez J, Soria E. Intervening sequences of regularly spaced prokaryotic repeats derive from foreign genetic elements. Journal of Molecular Evolution. 2005;60(2):174–182. 10.1007/s00239-004-0046-3 15791728

[14] Brouns SJJ, Jore MM, Lundgren M, Westra ER, Slijkhuis RJH, Snijders APL, et al. Small CRISPR RNAs guide antiviral defense in prokaryotes. Science. 2008;321(5891):960–4. 10.1126/science.1159689 18703739 PMC5898235

[15] Jinek M, Chylinski K, Fonfara I, Hauer M, Doudna JA, Charpentier E. A Programmable Dual-RNA—Guided DNA Endonuclease in Adaptice Bacterial Immunity. Science. 2012;337(August):816–822. 10.1126/science.1225829 22745249 PMC6286148

[16] Paez-Espino D, Morovic W, Sun CL, Thomas BC, Ueda Ki, Stahl B, et al. Strong bias in the bacterial CRISPR elements that confer immunity to phage. Nature Communications. 2013;4:1430. 10.1038/ncomms2440 23385575

[17] van Houte S, Ekroth AK, Broniewski JM, Chabas H, Ashby B, Bondy-Denomy J, et al. The diversity-generating benefits of a prokaryotic adaptive immune system. Nature. 2016;532(7599):385. 10.1038/nature17436 27074511 PMC4935084

[18] Bonsma-Fisher M, Soutière D, Goyal S. How adaptive immunity constrains the composition and fate of large bacterial populations. Proceedings of the National Academy of Sciences. 2018;115(32):E7462–E7468. 10.1073/pnas.1802887115 30038015 PMC6094150

[19] Toms A, Barrangou R. On the global CRISPR array behavior in class I systems. Biology direct. 2017;12(1):20. 10.1186/s13062-017-0193-2 28851439 PMC5575924

[20] Levin BR, Moineau S, Bushman M, Barrangou R. The Population and Evolutionary Dynamics of Phage and Bacteria with CRISPR-Mediated Immunity. PLoS Genetics. 2013;9(3). 10.1371/journal.pgen.1003312 23516369 PMC3597502

[21] Clauset A, Shalizi CR, Newman MEJ. Power-Law Distributions in Empirical Data. SIAM Review. 2009;51(4):661–703. 10.1137/070710111

[22] Mojica FJM, Díez-Villaseñor C, García-Martínez J, Almendros C. Short motif sequences determine the targets of the prokaryotic CRISPR defence system. Microbiology. 2009;155(3):733–740. 10.1099/mic.0.023960-0 19246744

[23] Sashital DG, Wiedenheft B, Doudna JA. Mechanism of Foreign DNA Selection in a Bacterial Adaptive Immune System. Molecular Cell. 2012;46(5):606–615. 10.1016/j.molcel.2012.03.020 22521690 PMC3397241

[24] Swarts DC, Mosterd C, van Passel MWJ, Brouns SJJ. CRISPR interference directs strand specific spacer acquisition. PLoS ONE. 2012;7(4). 10.1371/journal.pone.0035888 22558257 PMC3338789

[25] Staals RHJ, Jackson SA, Biswas A, Brouns SJJ, Brown CM, Fineran PC. Interference dominates and amplifies spacer acquisition in a native CRISPR-Cas system. Nature Communications. 2016;23:127–135. 10.1038/ncomms12853 PMC5059440 27694798

[26] Paez-Espino D, Sharon I, Morovic W, Stahl B, Thomas BC, Barrangou R, et al. CRISPR immunity drives rapid phage genome evolution in streptococcus thermophilus. mBio. 2015;6(2):1–9. 10.1128/mBio.00262-15 25900652 PMC4453577

[27] Westra E.R., Levin B.R. It is unclear how important CRISPR-Cas systems are for protecting natural populations of bacteria against infections by mobile genetic elements. Proceedings of the National Academy of Sciences. 2020; 117(45): 27777–27785 10.1073/pnas.1915966117 33122438 PMC7668106

[28] Hynes AP, Villion M, Moineau S. Adaptation in bacterial CRISPR-Cas immunity can be driven by defective phages. Nature Communications. 2014; 245:1–6. 25056268 10.1038/ncomms5399

[29] Mosterd C, Moineau S. Characterization of a type II-A CRISPR-Cas system in Streptococcus mutans. Msphere. 2020; 24;5(3) 10.1128/mSphere.00235-20 32581075 PMC7316486

[30] McGinn J. and Marraffini L.A., 2016. CRISPR-Cas systems optimize their immune response by specifying the site of spacer integration. Molecular cell, 2016, 64(3), 616–623. 10.1016/j.molcel.2016.08.038 27618488 PMC5096952

[31] Li M., Wang R., Zhao D. and Xiang H. Adaptation of the Haloarcula hispanica CRISPR-Cas system to a purified virus strictly requires a priming process. Nucleic Acids Research, 2014, 42(4), 2483–2492. 10.1093/nar/gkt1154 24265226 PMC3936756

[32] Datsenko KA, Pougach K, Tikhonov A, Wanner BL, Severinov K, Semenova E. Molecular memory of prior infections activates the CRISPR/Cas adaptive bacterial immunity system. Nature Communications. 2012;3(May):945. 10.1038/ncomms1937 22781758

[33] Rao C., Chin D. and Ensminger A.W. Priming in a permissive type IC CRISPR–Cas system reveals distinct dynamics of spacer acquisition and loss. RNA. 2017 23(10), 1525–1538. 10.1261/rna.062083.117 28724535 PMC5602111

[34] Kupczok A., Bollback J.P. Probabilistic models for CRISPR spacer content evolution. BMC evolutionary biology, 2013, 13(1), 54. 10.1186/1471-2148-13-54 23442002 PMC3704272

[35] Gudbergsdottir S., Deng L., Chen Z., Jensen J.V., Jensen L.R., She Q. and Garrett R.A. Dynamic properties of the Sulfolobus CRISPR/Cas and CRISPR/Cmr systems when challenged with vector-borne viral and plasmid genes and protospacers. Molecular microbiology, 2011, 79(1), 35–49. 10.1111/j.1365-2958.2010.07452.x 21166892 PMC3025118

[36] Watson BN, Vercoe RB, Salmond GP, Westra ER, Staals RH, Fineran PC. Type IF CRISPR-Cas resistance against virulent phages results in abortive infection and provides population-level immunity. Nature Communications. 2019 4;10(1):1–8. 10.1038/s41467-019-13445-2 31797922 PMC6892833

[37] Chen IMA, Markowitz VM, Chu K, Palaniappan K, Szeto E, Pillay M, et al. IMG/M: integrated genome and metagenome comparative data analysis system. Nucleic acids research. 2016;45(D1):D507–D516. 10.1093/nar/gkw929 27738135 PMC5210632

[38] Paez-Espino D, Chen IMA, Palaniappan K, Ratner A, Chu K, Szeto E, et al. IMG/VR: a database of cultured and uncultured DNA Viruses and retroviruses. Nucleic Acids Research. 2017;45(D1):D457. 10.1093/nar/gkw1030 27799466 PMC5210529

[39] Reddy TBK, Thomas AD, Stamatis D, Bertsch J, Isbandi M, Jansson J, et al. The Genomes OnLine Database (GOLD) v.5: a metadata management system based on a four level (meta)genome project classification. Nucleic Acids Research. 2015;43(D1):D1099–D1106. 10.1093/nar/gku950 25348402 PMC4384021

[40] Bland C, Ramsey TL, Sabree F, Lowe M, Brown K, Kyrpides NC, et al. CRISPR Recognition Tool (CRT): a tool for automatic detection of clustered regularly interspaced palindromic repeats. BMC Bioinformatics. 2007;8(1):209. 10.1186/1471-2105-8-209 17577412 PMC1924867

[41] Huntemann M, Ivanova NN, Mavromatis K, Tripp HJ, Paez-Espino D, Palaniappan K, et al. The standard operating procedure of the DOE-JGI Microbial Genome Annotation Pipeline (MGAP v.4). Standards in Genomic Sciences. 2015;10(1):86. 10.1186/s40793-015-0077-y 26512311 PMC4623924

[42] Benson DA, Cavanaugh M, Clark K, Karsch-Mizrachi I, Lipman DJ, Ostell J, et al. GenBank. Nucleic Acids Research. 2013;41(D1):D36–D42. 10.1093/nar/gks1195 27899564 PMC5210553

[43] Relman D.A. Microbial genomics and infectious diseases. New England Journal of Medicine. 2011, 365(4), 347–357. 10.1056/NEJMra1003071 21793746 PMC3412127

[44] Alstott J, Bullmore E, Plenz D. powerlaw: a Python package for analysis of heavy-tailed distributions. PloS ONE. 2014;9(1):e85777. 10.1371/journal.pone.0085777 24489671 PMC3906378

[45] Vuong Q.H., 1989. Likelihood ratio tests for model selection and non-nested hypotheses. Econometrica: Journal of the Econometric Society, 1989; 307–333. 10.2307/1912557

[46] Martynov A, Severinov K, Ispolatov I. Optimal number of spacers in CRISPR arrays. PLOS Computational Biology. 2017;13(12):1–23. 10.1371/journal.pcbi.1005891 29253874 PMC5749868

[47] Kagan YY. Earthquake size distribution: Power-law with exponent? Tectonophysics. 2010;490(1):103–114. 10.1016/j.tecto.2010.04.034

[48] Kolmogorov AN. Dissipation of Energy in the Locally Isotropic Turbulence. Proceedings of the Royal Society of London A: Mathematical, Physical and Engineering Sciences. 1991;434(1890):15–17.

[49] Newman MEJ. Power laws, Pareto distributions and Zipf’s law. Contemporary Physics. 2005;46(5):323–351. 10.1080/00107510500052444

[50] Clauset A, Young M, Gleditsch KS. On the Frequency of Severe Terrorist Events. Journal of Conflict Resolution. 2007;51(1):58–87. 10.1177/0022002706296157

[51] Gabaix X, Gopikrishnan P, Plerou V, Stanley HE. A theory of power-law distributions in financial market fluctuations. Nature. 2003;423:267–270. 12748636 10.1038/nature01624

[52] Beare B.K. and Toda A.A. On the Emergence of a Power Law in the Distribution of COVID-19 Cases. Physica D: Nonlinear Phenomena. 2020;412(132649). 10.1016/j.physd.2020.132649 PMC7365130 32834250

[53] Hatton IA, McCann KS, Fryxell JM, Davies TJ, Smerlak M, Sinclair ARE, et al. The predator-prey power law: Biomass scaling across terrestrial and aquatic biomes. Science. 2015;349(6252):aac6284–aac6284. 10.1126/science.aac6284 26339034

[54] Farrior CE, Bohlman SA, Hubbell S, Pacala SW. Dominance of the suppressed: Power-law size structure in tropical forests. Science. 2016;351(6269):2014–2016. 10.1126/science.aad0592 26744402

[55] Wiser MJ, Ribeck N, Lenski RE. Long-Term Dynamics of Adaptation in Asexual Populations. Science. 2013;342(6164):1364–1367. 10.1126/science.1243357 24231808

[56] Zhao K, Tseng BS, Beckerman B, Jin F, Gibiansky ML, Harrison JJ, et al. Psl trails guide exploration and microcolony formation in Pseudomonas aeruginosa biofilms. Nature. 2013;497(7449):388–91. 23657259 10.1038/nature12155 PMC4109411

[57] Meyer S, Held L, et al. Power-law models for infectious disease spread. The Annals of Applied Statistics. 2014;8(3):1612–1639. 10.1214/14-AOAS743

[58] Lloyd KG, Steen AD, Ladau J, Yin J, Crosby L. Phylogenetically Novel Uncultured Microbial Cells Dominate Earth Microbiomes. mSystems. 2018;3(5). 10.1128/mSystems.00055-18 30273414 PMC6156271

[59] Kallmeyer J, Pockalny R, Adhikari RR, Smith DC, D’Hondt S. Global distribution of microbial abundance and biomass in subseafloor sediment. Proceedings of the National Academy of Sciences. 2012;109(40):16213–16216. 10.1073/pnas.1203849109 22927371 PMC3479597

[60] Tiessen A., Pérez-Rodríguez P. and Delaye-Arredondo L.J. Mathematical modeling and comparison of protein size distribution in different plant, animal, fungal and microbial species reveals a negative correlation between protein size and protein number, thus providing insight into the evolution of proteomes. BMC research notes, 2012, 5(1), p.85. 10.1186/1756-0500-5-85 22296664 PMC3296660

[61] Hong Yue-Hui and Deng Mao-Cheng and Xu Xiao-Ming and Wu, et al. Characterization of the transcriptome of Achromobacter sp. HZ01 with the outstanding hydrocarbon-degrading ability. Gene. 2016;2(584):185–194. 10.1016/j.gene.2016.02.032 26915487

[62] Simon HA. On a class of skew distribution functions. Biometrika. 1955;42(3/4):425–440. 10.2307/2333389

[63] Bradde S., Nourmohammad A., Goyal S. and Balasubramanian V. The size of the immune repertoire of bacteria. Proceedings of the National Academy of Sciences, 2020, 117(10), 5144–5151. 10.1073/pnas.1903666117 32071241 PMC7071851

[64] Turkington C.J., Morozov A., Clokie M.R.J., Bayliss C.D. Phage-resistant phase-variant sub-populations mediate herd immunity against bacteriophage invasion of bacterial meta-populations. Frontiers in Microbiology. 2019; 10, 1473. 10.3389/fmicb.2019.01473 31333609 PMC6625227

[65] De Los Rios P, Zhang YC. Universal 1/f noise from dissipative self-organized criticality models. Physical Review Letters. 1999;82(3):472. 10.1103/PhysRevLett.82.472

[66] Patterson AG, Jackson SA, Taylor C, Evans GB, Salmond GPC, Przybilski R, et al. Quorum Sensing Controls Adaptive Immunity through the Regulation of Multiple CRISPR-Cas Systems. Molecular Cell. 2016; p. 1–7. 10.1016/j.molcel.2016.11.012 27867010 PMC5179492

[67] Benton MJ. The Red Queen and the Court Jester: species diversity and the role of biotic and abiotic factors through time. Science. 2009;323(5915):728–732. 10.1126/science.1157719 19197051

[68] Haerter JO, Sneppen K. Spatial structure and Lamarckian adaptation explain extreme genetic diversity at CRISPR locus. MBio. 2012 31;3(4). 10.1128/mBio.00126-12 22807565 PMC3413401

[69] Pyenson N.C. and Marraffini L.A. Co-evolution within structured bacterial communities results in multiple expansion of CRISPR loci and enhanced immunity. Elife, 9, p.e53078. 10.7554/eLife.53078 32223887 PMC7105378

