# Supplementary material for: Searching for fat tails in CRISPR-Cas systems: Data analysis and mathematical modeling
Source: PLoS Comput Biol. 2021 Mar 26;17(3):e1008841. doi: 10.1371/journal.pcbi.1008841 (PMC8026048; doi:10.1371/journal.pcbi.1008841)
Supplement: S1 Text — Fig A. Flowchart of the mathematical model presenting possible outcomes of bacteria-phage interaction and replication for bacteria having i spacers. Fig B. Schematic representation of transition between size classes (containing different spacer numbers i ) at time iteration t. Fig C. Temporal evolution of the transition matrix eigenvalues. Fig D. Statistical distribution of spacers in CRISPR array predicted by the model with constant qi and νi. Fig E. Introducing parameter dependence on the CRISPR array length i. Fig F. Spacer distribution for linear dependence of qi and νi affected by variation of Sl and μn. Fig G. Sensitivity of α with respect to variation of SL (the fraction of spacers lost during replication) and s (the probability of survival if the microbe has no spacer for the virus). Fig H. Sensitivity of α with respect to variation of μn (the average number of spacers gained) and s (the probability of survival if the microbe has no spacer for the virus). Fig I. Sensitivity of α with respect to variation of h < 1 (the probability of protospacer match), g (the probability of survival in the case of mutated protospacer) and s. s ≤ g ≤ h. Fig J. Variation of statistical distribution of spacers (linear qi and νi ) for different g (the probability of survival in the case of mutated protospacer). Fig K. Variation of statistical distribution of spacers (linear qi and νi ) for different parameters s, p, μm, μk and h. Table A. Comparison of the Truncated Power Law p(x)∼x−α e−λx to other heavy-tailed candidate distributions in fitting empirical distribution of spacers for combined metagenomes from samples collected in different environments. (PDF) [file pcbi.1008841.s001.pdf]

Supplementary information for
Searching for Fat Tails in CRISPR-Cas Systems: Data Analysis and Mathematical Modeling

Yekaterina S. Pavlova[1], David Paez-Espino[2,3], Andrew Yu. Morozov[*,4,5], Ilya S. Belalov[*,6],

1 Palomar College, Mathematics Department, San Marcos, California, United States of America
2 Department of Energy, Joint Genome Institute, Walnut Creek, California, United States of America
3 Mammoth BioSciences, South San Francisco, California, United States of America
4 School of Mathematics, University of Leicester, Leicester, United Kingdom
5 Institute of Ecology and Evolution, Russian Academy of Sciences, Moscow, Russia
6 Laboratory of Microbial Viruses, Winogradsky Institute of Microbiology, Research Center of Biotechnology, Russian Academy of Sciences, Moscow, Russia

* am379@leicester.ac.uk (AM) ; ilya.belalov@gmail.com (IB)

Constructing Mathematical Model

We consider a bacterial population subjected to viral infection. The total population size is assumed to be constant: its level is set at the carrying capacity of the environment. The model does not discriminate between different types of spacers or CRISPRs. In essence, we assume that a single CRISPR array represents an individual bacterial or archaeal cell. Our main goal is to reveal the long term stationary distribution of spacers in the bacterial population.

The system is modeled as a discrete-time Markov chain (multi-type branching process) with the state vector $\mathbf{F}(t) = \langle F_1(t), ..., F_N(t)\rangle$, where $F_i(t)$ is the number of microbes having i spacers at time $t \in \mathbb{Z}_{\geq 0}$. We assume that the maximal number of spacers that the bacteria may have cannot exceed some fixed large number N. This is related to the fact that having and using the CRISPR-Cas system results in fitness costs [1, 2].

At each iteration, the class sizes F_i are updated depending on probabilistic proportions of bacteria leaving the class (death), bacteria staying in the same class (no loss or gain of new spacers) and bacteria arriving from a lower (gain of spacers due to virus infection) and an upper (replication with loss of spacers) classes (S1 Fig B).

Each time iteration t is split into two stages which are represented in S1 Fig A and S1 Fig B. During the first stage (S1 Fig A(i)) we consider the events related to virus-host interactions (after the virus met the host) resulting in death or survival with a gain of spacers or keeping their number the same. We use $F_i(t + \frac{1}{2})$ to denote the mid-iteration class sizes. The proportion of microorganisms that die during the first stage is then taken into account in the second stage (S1 Fig A(ii)) to determine the scaling for replication rate so that the same population size is always maintained. $F_i(t + 1)$ denote the class sizes at the end of the second stage.

First Half-Iteration. Infection by the phage

The corresponding flowchart is given by S1 Fig A(ii).

In order for bacterial or archaeal cell to die or gain spacers, it is necessary that it is infected by a virus (**Meet Virus**). In the model, this happens with probability p which is assumed to be constant: p represents a hypothetical viral load averaged over the Earth. In the model, meeting a virus by the cell signifies infection. The bacterium can have a spacer (**Has a Spacer**) corresponding to the given virus with the probability q_i. Since having a large collection of spacers (large i) generally increases the probability of having an appropriate one when a virus is encountered, we assume that q_i will generally be a monotonically increasing function of i.

There are different possible parameterisations of q_i. Here we mostly consider the simplest case of linear dependence given by

$$q_i = \frac{i-1}{N}. \tag{1}$$

Note that the above approximation will be suitable if we assume that each new spacer added to the CRISPR array is distinct until we reach the maximum amount N of distinct spacers. This may be a good approximation for bacteria having few spacers and exposed to a largely diverse viruses.

More realistically, when the CRISPR array gains more and more spacers, there will be a higher probability to have identical spacers to the ones already present in the CRISPR library. In the case i large enough, there will be almost no new distinct spacers to gain. On the other hand, the same scenario may occur for different phages, as well as for multitude of mutants of a single phage species. In this case, a more appropriate approximation may be a logistic function $q_i = \frac{1}{1+e^{-r(i-I)}}$, where the constant r determines the steepness of the curve and I is the number of spacers for which $q_I = \frac{1}{2}$ (the cell has a half of the distinct spacers present in the population). In our numerical simulations, we use both linear and logistic parameterisation of q_i.

Overall, the outcome for the cell having an appropriate spacer against the virus depends on the state of the viral protospacer and intrinsic efficiency of spacer. We assume that with probability h the protospacer matches the perfect spacer and the microbial cell is immune to the infection (BIM Bacteriophage-Immune Mutant). In this case, the cell either gains more spacers or persist without any new additions. The value of h generally depends on the equilibrium concentration of CEM (CRISPR-Escape Mutant) viruses in the system and overall efficiency of spacers. We assume that h is approximately constant. In the case of a spacer of poor quality (either due to mutation in PAM and/or protospacer, location at the trail-end of the CRISPR array, or low affinity to the effector Cas proteins), the cell either acquires immunity by gaining new spacers or it fails to do so and eventually dies. We assume that in this case, the cell survives with probability g.

In the case where the cell does not have a spacer for the virus it may still survive and gain new spacers *via* naive adaptation machinery with the probability s or die with the probability $1 - s$. The probability of survival g for a cell with an imperfect spacer should be larger then in the case the cell does not have spacer at all (given by s). Here and later a "bad spacer" refers to a spacer which does not perfectly protect from virus.

Having met the virus, there are three possibilities to gain spacers (see S1 Fig A). The model assumes that in all three cases the number of spacers added is exponentially distributed with the mean μ_n for immune cells (**Good spacer**), μ_m for cells which are not guaranteed survival (**Bad spacer**), and μ_k for cells that do not have a spacer(**No Spacer**). The average number of spacers added to the CRISPR array having a bad

spacer μ_m is generally much larger (about two orders of magnitude) than in the case where the cell has no spacer (μ_k) [3]; in our numerical simulations we consider that $\mu_n/\mu_k = 100$. The average number of spacers added to the CRISPR of immune cells μ_n is only slightly larger (i.e. of the same order of magnitude) then μ_m [4]. In most simulations, we assume that they are equal, however, we also numerically test the dependence of a final spacer distribution on the difference of μ_n and μ_m (S1 Fig K(iv)). Although, acquisition of many spacers during a single infection event to the best of our knowledge was not demonstrated in experiments thus far, the model reflects natural spacer acquisition for two unrelated reasons. The infection related half-iteration may be viewed as subsequent encountering of several phages and collecting spacers from them. The second reason follows from the scale-invariance of the distribution of spacers in CRISPR arrays (Figs 2b and 3 in the main text). Scale-invariance implies identical behaviour of subsystems to the entire system, *e.g.* curve of the distribution of spacers per single CRISPR array has the same shape as the distribution of spacers per bacterial colony, which is the same as distribution of spacers per tidepool, and so forth. The known parameter relationships are summarized in S1 Fig A.

The probability of adding $n > 0$ spacers with an exponential distribution and the mean μ is given by

$$P(\mu, n) = \begin{cases} \int_0^1 \frac{1}{\mu} e^{-\frac{x}{\mu}} dx = 1 - e^{-1/\mu} & \text{if } n = 1, \\ \int_{n-1}^{n} \frac{1}{\mu} e^{-\frac{x}{\mu}} dx = e^{-(n-1)/\mu} - e^{-n/\mu} & \text{if } n > 1. \end{cases} \quad (2)$$

Consider that at time t the cell had i spacers. The probabilities of a cell having i spacers to die D_i and to gain extra $j - i > 0$ spacers $Q_{i,j}$ are given, respectively, by

$$D_i = P(X_{t+\frac{1}{2}} = \text{Death} | X_t = i) = p\left[q_i(1-h)(1-g) + (1-q_i)(1-s)\right] \quad (3)$$

$$Q_{i,j} = P(X_{t+\frac{1}{2}} = j > i \,| X_t = i) =$$

$$p\left[q_i\left\{hP(\mu_n, j-i) + (1-h)gP(\mu_m, j-i)\right\} + (1-q_i)sP(\mu_k, j-i)\right] \quad (4)$$

Although explicit experimental demonstration of acquisition of more than one spacer at a time is still to be done, there is some empirical support of this. For example, in experiments by [6], one can see acquisition of 5-6 spacers; in other studies acquisition of up to 4 spacers was reported by the end of the experiment [4, 7]. Although, in the two latter papers the reported number of spacers > 1 can be explained by the fact that this was observed after several replication cycles, it is well possible that in some bacteria priming resulted in > 1 spacers. However, even in this case, this empirical evidence would be suitable for justification of our conceptual model. Indeed, by a single (combined) infection cycle in our mathematical model we can understand several physical infection cycles combined together. The type of resultant curve of the distribution of spacers will be qualitatively the same as for the single one. Finally, the scale invariance of the distribution of spacers and the observed power law of spacers within individual metagenomes (see Fig 2 in the main text) should potentially allow us to be able to consider the distribution of spacers in single arrays in small ecosystems to be equivalent to the one observed in larger ecosystems (per bacterial colony).

For a low or intermediate i, the probability $Q_{i,j}$ decays exponentially with the number of gained spacers $j - i$. However, for i close to the maximum class size N, the fraction of bacteria which try to add more then the maximum number of spacers becomes no longer be negligible. In reality, there is no restriction for bacteria to always add spacers regardless of the current length of the CRISPR array, although the maximum number of spacers observed in empirical data is of the order $10^2 - 10^4$ (see Fig 1a. in the main text). In our model, we can account for the possibility of adding

any number of spacers. We proceed in the following way. We allow the microbial cell to add spacers to go beyond N. However, we assume that such individuals will not be able to replicate, since combined the costs of replication and operating long CRISPR array are too high which will cause the death of the cell. As we discuss in the next subsection, the cell is only able to lose spacers during DNA replication and since cells from high spacer content classes F_i ($i \geq N$) do not replicate, they will not influence lower classes by supplying them with new members. As such, for each class F_i, a small fraction of bacteria that will leave the maximal spacer range which is described by

$$Q_{i,(j>N)} = p\left[q_i\left\{he^{-\frac{(N-i)}{\mu_n}} + (1-h)ge^{-\frac{(N-i)}{\mu_m}}\right\} + (1-q_i)se^{-\frac{(N-i)}{\mu_k}}\right]. \quad (5)$$

In our numerical simulations, the scale of the problem (the maximum spacers number N and the total population size) is set up so that the highest nonempty class F_i has $i < N$ or $F_i \ll 1$ for i close to N, and thus the boundary condition at $i = N$ does not affect the stationary distribution of spacers. However, in order to avoid amplification of small losses of those cells after a large number of iterations and maintain a constant population size within the first N classes, we include all such bacteria into the death record to keep the population size constant (see next subsection for detail).

The total fraction of class $F_i(t)$ that stays unchanged at the first-half iteration is described by $1-p$ which does not depend on i. In other words, the only microbes to maintain their spacer content are the ones that do not meet the virus. Having met the virus, the cell either dies or gains a non-zero amount of new spacers. In the first half-iteration, classes F_i may receive new members only from lower classes F_n with $n = 1, 2, ..., i-1$ through spacer addition, thus for the half-iteration point we get

$$F_1\left(t+\frac{1}{2}\right) = (1-p)F_1(t),$$

$$F_i\left(t+\frac{1}{2}\right) = \sum_{n=1}^{i-1} Q_{n,i}F_n(t) + (1-p)F_i(t),$$

$$F_N\left(t+\frac{1}{2}\right) = \sum_{n=1}^{N-1} Q_{n,N}F_n(t) + (1-p)F_N(t),$$

which gives us the following transition matrix $\mathbf{A}^{(1)}$ describing the first half-iteration

$$\mathbf{A}^{(1)} = \begin{pmatrix} (1-p) & Q_{1,2} & Q_{1,3} & Q_{1,4} & ... & Q_{1,N} \\ 0 & (1-p) & Q_{2,3} & Q_{2,4} & ... & Q_{2,N} \\ 0 & 0 & (1-p) & Q_{3,4} & ... & Q_{3,N} \\ 0 & 0 & 0 & (1-p) & ... & Q_{4,N} \\ . & . & . & . & . & . \\ 0 & 0 & 0 & 0 & ... & (1-p) \end{pmatrix}. \quad (6)$$

The half-iteration class sizes can be computed by matrix multiplication

$$\mathbf{F}\left(t+\frac{1}{2}\right) = \mathbf{F}(t)\mathbf{A}^{(1)}. \quad (7)$$

Second Half-Iteration. Replication

We assume that the bacterial population has settled at a constant total size allowed by the carrying capacity of the environment. Since during the first half-iteration some cells die, we need to compensate for the loss with new replication events. Microbes

with shorter CRISPRs generally reproduce faster. Thus, the replication rate of bacteria ν_i depends on i, the number of spacers present. Various parameterisations of ν_i are possible. Here we mostly focus on the simplest case which is the linear dependence

$$\nu_i = 1 - \frac{i-1}{N}. \tag{8}$$

Spacers in microbial genomes generally get lost during replication. Bacteria having i spacers can not lose more than $i-1$, so we consider that the loss of more than $i-1$ spacers (in mathematical expressions for probabilities) is equivalent to placing bacteria in the class F_1. Due to the nature of DNA replication process, it is likely that longer CRISPR arrays suffer larger losses and in general we can expect that the average number of spacers lost $\mu_\delta(i)$ depends on the number of spacers that the cell has. Here we parametrise this dependence by setting $\mu_\delta(i) = S_L i$, where S_L is the fraction of the total spacers in CRISPR array that may be deleted from the newly synthesized DNA strand. Then the probabilities of deletion of $i-j$ spacers from a daughter cell having i spacers are given by

$$\begin{cases} L_{1,1} = 1, & \text{no spacer loss in } F_1 \text{ cells,} \\ L_{i,1} = e^{-\frac{(i-1)}{\mu_\delta(i)}}, & \text{if } i = 2, ..., N \text{ lose all spacers,} \\ L_{i,j} = e^{-\frac{(i-j)}{\mu_\delta(i)}} - e^{-\frac{(i-j+1)}{\mu_\delta(i)}}, & \text{if } i = 2, ..., n,\ j = 2, ...i-1, \\ L_{i,i} = 1 - e^{-\frac{1}{\mu_\delta(i)}}, & \text{no spacer loss.} \end{cases} \tag{9}$$

Note that even though $\mu_\delta(i)$ varies with i, for any fixed i the sum of probabilities of all the outcomes adds up to unity.

$$\sum_{j=1}^{i} L_{i,j} = e^{-\frac{(i-1)}{\mu_\delta(i)}} + (e^{-\frac{(i-2)}{\mu_\delta(i)}} - e^{-\frac{(i-1)}{\mu_\delta(i)}}) + ... + (1 - e^{-\frac{1}{\mu_\delta(i)}}) = 1. \tag{10}$$

In the process of genomic DNA replication the replication fork is formed by the leading and lagging strands. The former is synthesized by a single polymerase particle moving to the 5'-end of the copied DNA strand and, thus it is hardly expected to proceed to template switch or collide with DNA hairpins. However, the latter (the lagging strand) is base-paired to dozens of Okazaki fragments. The loop structure of the lagging strand naturally provides opportunities for template switch by multiple polymerase particles synthetizing Okazaki fragments and for recombination with the help of DNA reparation enzymes also present in the loop of the lagging strand. We assume that exactly half of the new daughter cells maintain their spacer content (the ones inheriting the leading DNA strand), therefore the total fraction of the half-iteration population $F_i(t+\frac{1}{2})$ that remains in F_i at the end of the iteration is

$$\begin{cases} S_1 = 1 + N_\nu \nu_i, & \text{all } F_1 \text{ daughter cells stay in } F_1 \\ S_i = 1 + N_\nu \nu_i L_{i,i}, & i = 2, ...N \end{cases} \tag{11}$$

where N_ν is the scaling factor needed to ensure the constant microbial population size. All new cell divisions must balance all the departures from the system. Summing up all above contributions and equating them to the total number of all new replication events we get

$$\sum_{i=1}^{N} \left[D_i + p \left\{ q_i(he^{-\frac{(N-i)}{\mu_n}} + (1-h)ge^{-\frac{(N-i)}{\mu_m}}) + (1-q_i)se^{-\frac{(N-i)}{\mu_k}} \right\} \right] F_i(t) = \\ = \sum_{i=1}^{N} N_\nu \nu_i F_i(t+\frac{1}{2}). \tag{12}$$

Using the mid-iteration class sizes $F_i(t+\frac{1}{2})$ already computed at the half-iteration point, the scaling factor N_ν can easily be computed as

$$N_\nu = \frac{\sum_{i=1}^{N}\left[D_i + p\left\{q_i(he^{-\frac{(N-i)}{\mu_n}} + (1-h)ge^{-\frac{(N-i)}{\mu_m}}) + (1-q_i)se^{-\frac{(N-i)}{\mu_k}}\right\}\right]F_i(t)}{\sum_{i=1}^{N}\nu_i F_i(t+\frac{1}{2})}. \tag{13}$$

The class F_i increases its density due to inflow from higher classes F_n with $n = i+1, i+2, ..., N$, which lose their spacers during replication (see S1 Fig A and S1 Fig B). Let $\Delta_{i,j}$ be the probability of the class F_i to lose $i-j$ spacers during replication. For $\Delta_{i,j}$ we have

$$\Delta_{i,j} = N_\nu \nu_i L_{i,j}.\,, \quad j = i-1, ..., 2, 1 \tag{14}$$

We can now write down the following transition equations to calculate the state of the system at the end of the second half-iteration

$$F_1(t+1) = S_1 F_1(t+\frac{1}{2}) + \sum_{n=2}^{N}\Delta_{n,1}F_n(t+\frac{1}{2}),$$

$$F_i(t+1) = S_i F_i(t+\frac{1}{2}) + \sum_{n=i+1}^{N}\Delta_{n,i}F_n(t+\frac{1}{2}),$$

$$F_N(t+1) = S_N F_N(t+\frac{1}{2}),$$

which gives us the second half-iteration transition matrix $\mathbf{A}^{(2)}$ for the reproduction of the population

$$\mathbf{A}^{(2)} = \begin{pmatrix} S_1 & 0 & 0 & 0 & .. & 0 \\ \Delta_{2,1} & S_2 & 0 & 0 & ... & 0 \\ \Delta_{3,1} & \Delta_{3,2} & S_3 & 0 & ... & 0 \\ \Delta_{4,1} & \Delta_{4,2} & \Delta_{4,3} & S_4 & ... & 0 \\ . & . & . & . & . & . \\ \Delta_{N,1} & \Delta_{N,2} & \Delta_{N,3} & \Delta_{N,4} & ... & S_N \end{pmatrix}. \tag{15}$$

The update of class sizes at the end of the second half-iteration can be computed multiplication of the transition matrices

$$\mathbf{F}(t+1) = \mathbf{F}\Big(t+\frac{1}{2}\Big)\mathbf{A}^{(2)}. \tag{16}$$

This completes one-step transition.

Equilibrium Probability Distribution

Each consecutive iteration is computed by multiplication of the above matrices $\mathbf{A}^{(1)}$ and $\mathbf{A}^{(2)}$. The one-step transition matrix $\mathbf{A} = \mathbf{A}^{(1)}\mathbf{A}^{(2)}$ is irreducible since $A_{i,j} \neq 0$ for all $i, j = 1, ..., N$ (all spacer content states are accessible from all other states in one step). The matrix is also aperiodic since all states have period 1 by construction. By the convergence theorems for finite state space discrete time Markov Chain theory [5]p163 an irreducible aperiodic Markov chain is guaranteed to have a unique limiting probability distribution $\mathbf{F}^*$ that is independent of initial conditions $\mathbf{F}(0)$.

$$\mathbf{F}^* = \lim_{t\to\infty} (\mathbf{F}(0)\mathbf{A}^{(1)}\mathbf{A}^{(2)})^t = \lim_{t\to\infty} \mathbf{A}^t\mathbf{F}(0) \tag{17}$$

We used MATLAB software to compute the first ($\max(\lambda_{\mathbf{A}})$) and second ($\lambda^r_{\mathbf{A}}$) largest eigenvalues of the transition matrix $\mathbf{A}$ (S1 Fig C). The replication scaling coefficient N_ν was adjusted at each iteration in order to preserve the constant population density. The value of $\max(\lambda_{\mathbf{A}})$ settles to 1 as expected, and $\lambda^r_{\mathbf{A}} < 1$ determines the convergence rate that will vary depending on parameter values (S1 Fig C). The equilibrium distribution $\mathbf{F}^*$ is the principal eigenvector of the transition matrix $\mathbf{A}$ corresponding to $\max(\lambda_{\mathbf{A}})= 1$ and thus $\mathbf{F}^*$ is invariant regarding the transition matrix $\mathbf{A}$, i.e.

$$\mathbf{F}^* = \mathbf{F}^*\mathbf{A}. \tag{18}$$

To find $\mathbf{F}^*$, we conducted a large number of direct iterations starting from initial conditions based on the matrix $\mathbf{A}$. We also computed $\mathbf{F}^*$ from equation (18). The results of both methods were identical (see next section).

Results of Model Simulation

The maximum number of spacers was $N = 1,000$. We started from a uniform initial distribution $F_i(0) = 1,000$ for $i = 1, ..., N$, as well as point-wise distribution $F_1(0) = 1,000,000$, $F_i(0) = 0$ for $i = 2, ..., N$ (initially all bacteria in the population have a minimal amount of spacers, reside in class F_1). In both cases, the resultant distributions of spacers were the same.

Stationary class size distribution $\mathbf{F}^*$ was attained on average in less then $4,000$ iterations (maximum $10,000$) and was independent of the initial conditions. When a power law distribution of class sizes was attained, the maximum number of spacers observed i_{max} was ranging from around 100 to 1000 spacers (nonempty classes $F_1 - F_{i_{max}}$). For a larger range of nonempty classes, we need to consider larger initial population sizes which in general require larger values of maximal size N. This becomes computationally very expensive while producing the same distribution type for the same parameter values.

We first test our model with constant probability parameter values $q_i = q$ and $\nu_i = 1$ (S1 Fig D.), i.e. with constant CRISPR efficiency and the replication rates. The obtained stationary distribution $\mathbf{F}^*$ is either normal or skew normal. The average number i computed using numerical simulation data increases with an increase in μ_n (signifying a larger average spacer gain) and decreases S_L (smaller fractions of existing spacers are lost during replication). Considering a linear dependence on i of the probability to have a spacer q_i results in right-centered distribution of normal type (S1 Fig E(i)). In the case where q_i is kept constant while the replication rate decreases with i, we get a left-centered distribution of spacers, however the corresponding distribution is still of normal/Gaussian type (S1 Fig E(ii,iii,iv)). The fat tail property characteristic to the power law distribution is only achieved in this model when we allow both q_i and ν_i to depend on i (S1 Fig E(v)).

Considering a linearly increasing q_i combined with a linear decreasing ν_i generates a left-centered fat tail distribution provided the parameter μ_n is within a certain range which depends on the value of S_L (S1 Figs F, J, K). For μ_n too being large, excessive spacer addition pushes the distribution away from left-centeredness and for too small μ_n, almost all cells cluster in the lowest class F_1. We used **python powerlaw** package to carry out statistical analysis of numerical simulation results. Since we are primarily interested in identifying parameter values that would generate a power law distribution, we test the likely heavy-tailed candidates using powerlaw package maximum likelihood analysis utility against lognormal, exponential, stretched

exponential, truncated power law, and power law distributions. The minimum likelihood values and maximum p-values are labeled on the graphs. We find that a truncated power law is the best fit for almost all cases where left-centered heavy tailed distribution is observed.

Decreasing S_L (S1 Fig F(i,ii)) or increasing μ_n (S1 Fig F(iii,iv)) generally results in decreasing the power law exponent α (S1 Fig F(i,ii)) and also increasing the number i_{max} of nonempty classes F_i. Admissible parameter values S_L and μ_n for different values of s are graphed in S1 Figs G, H. Larger probabilities of survival in the case of no spacer s in the cell generally extends the ranges of S_L and μ_n that generate a power law fit. In order to obtain the same α for a larger s, S_L has to be lowered (i.e. less spacers are lost during replication) or to get μ_n increased (more spacers are gained as a result of meeting a virus).

In the above figures, we consider the situation perfect CRISPR efficiency, i.e. $h = 1$. We also tested the case where CRISPR may be malfunctioning ($h < 1$): we considered the biologically relevant parameter ranges $s \leq g \leq h$ (S1 Fig I). Increasing g within this interval generally leads to a decrease of α; however the rate of decrease is very slow or negligible for the parameter values that generate $\alpha > 2$ within the interval $s \leq g \leq h$. Small variations in α is such cases are predominantly due to the fitting algorithm choice of $i_{min} \in [1, 15]$ and λ (S1 Fig J). Larger values of s (for fixed g and h) and smaller values of h (for fixed g and s) resulted in increased values of α and all cases (S1 Figs I, J, and K(i-v)).

Decreasing probability of meeting the virus p has a similar effect as decreasing g. In this case, α increases rapidly within some small range of values p after which a further decrease in p does not generate significant changes in α (S1 Fig K(ii)). Decreasing μ_m below μ_n (on average less spacers are acquired in the case of the bad spacer *versus* good spacer), while keeping other parameters fixed, produces similar effects as decreasing $\mu_n = \mu_m$, i.e. α increases and i_{max} decreases (S1 Fig K(iii)). An increase in the average number of spacers acquired in the case of no spacer μ_k shows a small increase in α but only when μ_k is within one order of magnitude of μ_n (S1 Fig K(iv)). Decreasing the probability of a perfect spacer h results in increasing α, however also only within some small range, otherwise α does not change significantly with decreasing h (S1 Fig K(v)).

References

1. Paez-Espino D, Morovic W, Sun CL, Thomas BC, Ueda Ki, Stahl B, et al. Strong bias in the bacterial CRISPR elements that confer immunity to phage. Nature Communications. 2013;4:1430. doi:10.1038/ncomms2440
2. Levin BR, Moineau S, Bushman M, Barrangou R. The Population and Evolutionary Dynamics of Phage and Bacteria with CRISPR-Mediated Immunity. PLoS Genetics. 2013;9(3). doi:10.1371/journal.pgen.1003312.
3. Datsenko KA, Pougach K, Tikhonov A, Wanner BL, Severinov K, Semenova E. Molecular memory of prior infections activates the CRISPR/Cas adaptive bacterial immunity system. Nature Communications. 2012;3(May):945. doi:10.1038/ncomms1937.
4. Staals RHJ, Jackson SA, Biswas A, Brouns SJJ, Brown CM, Fineran PC. Interference dominates and amplifies spacer acquisition in a native CRISPR-Cas system. Nature Communications. 2016;23:127–135. doi:10.1038/ncomms12853.
5. Privault N. Understanding Markov Chains. Springer; 2018.
6. Li, M., Wang, R., Zhao, D., Xiang, H. Adaptation of the Haloarcula hispanica CRISPR-Cas system to a purified virus strictly requires a priming process. Nucleic Acids Research, 2014; 42(4): 2483–2492. doi:10.1093/nar/gkt1154
7. Semenova E, Savitskaya E, Musharova O, Strotskaya A, Vorontsova D, Datsenko KA, Logacheva MD, Severinov K. Highly efficient primed spacer acquisition from targets destroyed by the Escherichia coli type I-E CRISPR-Cas interfering complex. PNAS 2016; 113(27): 7626–31. doi:10.1073/pnas.1602639113.

Table A. Comparison of the Truncated Power Law $p(x) \sim x^{-\alpha} e^{-\lambda x}$ to other heavy-tailed candidate distributions in fitting empirical distribution of spacers for combined metagenomes from samples collected in different environments. Positive Logikelyhood ratio indicates better fit for the Truncated Power Law distribution.

Metagenome Environment	Number of CRISPRs	α	λ	Candidate distribution	Loglikelihood ratio	P-value
Terrestrial(soil)	463532	2.609	0.0052	lognormal	$3.69x10^{1}$	$3.68x10^{-11}$
				exponential	$1.01x10^{3}$	$1.60x10^{-63}$
				stretched_exponential	$1.59x10^{1}$	$8.82x10^{-9}$
				power_law	$1.24x10^{1}$	$6.32x10^{-7}$
Engineered	229470	2.363	0.0064	lognormal	$6.09x10^{2}$	$2.67x10^{-132}$
				exponential	$1.49x10^{4}$	0.0
				stretched_exponential	$1.71x10^{2}$	$2.51x10^{-105}$
				power_law	$2.17x10^{2}$	0.0
Host(other)	131590	2.353	0.0065	lognormal	$7.28x10^{1}$	$1.15x10^{-18}$
				exponential	$2.03x10^{3}$	$7.63x10^{-122}$
				stretched_exponential	$2.44x10^{1}$	$1.15x10^{-16}$
				power_law	$3.18x10^{1}$	$1.44x10^{-15}$
Thermal Springs	219769	2.261	0.0105	lognormal	$6.00x10^{1}$	$1.03x10^{-13}$
				exponential	$5.07x10^{3}$	$6.09x10^{-260}$
				stretched_exponential	$3.56x10^{1}$	$7.13x10^{-10}$
				power_law	$3.07x10^{2}$	0.0
Freshwater	360441	2.78	0.0004	lognormal	$1.02x10^{3}$	$7.24x10^{-98}$
				exponential	$1.89x10^{4}$	$7.63x10^{-143}$
				stretched_exponential	$8.62x10^{2}$	$1.94x10^{-1}$
				power_law	3	$1.43x10^{-2}$
Non-marine Saline and Alkaline	121545	2.35	0.0098	lognormal	$2.36x10^{2}$	$6.97x10^{-47}$
				exponential	$6.42x10^{3}$	$3.77x10^{-301}$
				stretched_exponential	$6.02x10^{1}$	$5.47x10^{-35}$
				power_law	$1.58x10^{2}$	0.0
Host(human)	11161	3.688	0.0118	lognormal	$1.42x10^{1}$	$1.21x10^{-2}$
				exponential	$1.49x10^{2}$	$4.65x10^{-8}$
				stretched_exponential	2.88	$2.39x10^{-1}$
				power_law	$3.65x10^{-1}$	$3.93x10^{-1}$
Air	122	1.919	0.1004	lognormal	$6.59x10^{-3}$	$9.74x10^{-1}$
				exponential	2.05	$4.77x10^{-1}$
				stretched_exponential	$5.21x10^{-3}$	$9.63x10^{-1}$
				power_law	$4.42x10^{-1}$	$3.47x10^{-1}$
Terrestrial(other)	11044	1.905	0.0075	lognormal	$1.17x10^{1}$	$1.62x10^{-3}$
				exponential	$5.90x10^{2}$	$3.51x10^{-36}$
				stretched_exponential	2.73	$2.73x10^{-2}$
				power_law	9.86	$8.95x10^{-6}$
Aquatic Sediment	54540	3.222	5E-07	lognormal	4.79	$1.45x10^{-1}$
				exponential	$7.89x10^{1}$	$6.16x10^{-6}$
				stretched_exponential	1.85	$3.50x10^{-1}$
				power_law	$7.93x10^{-2}$	$6.90x10^{-1}$
Host(plant)	211746	2.631	0.0024	lognormal	$6.48x10^{1}$	$3.35x10^{-11}$
				exponential	$1.37x10^{3}$	$9.74x10^{-24}$
				stretched_exponential	$6.06x10^{2}$	$2.20x10^{-48}$
				power_law	4.39	$3.04x10^{-3}$
Marine	374143	2.898	0.0044	lognormal	$1.23x10^{2}$	$3.76x10^{-17}$
				exponential	$2.49x10^{3}$	$6.45x10^{-103}$
				stretched_exponential	$4.00x10^{1}$	$1.66x10^{-7}$
				power_law	8.42	$4.05x10^{-5}$

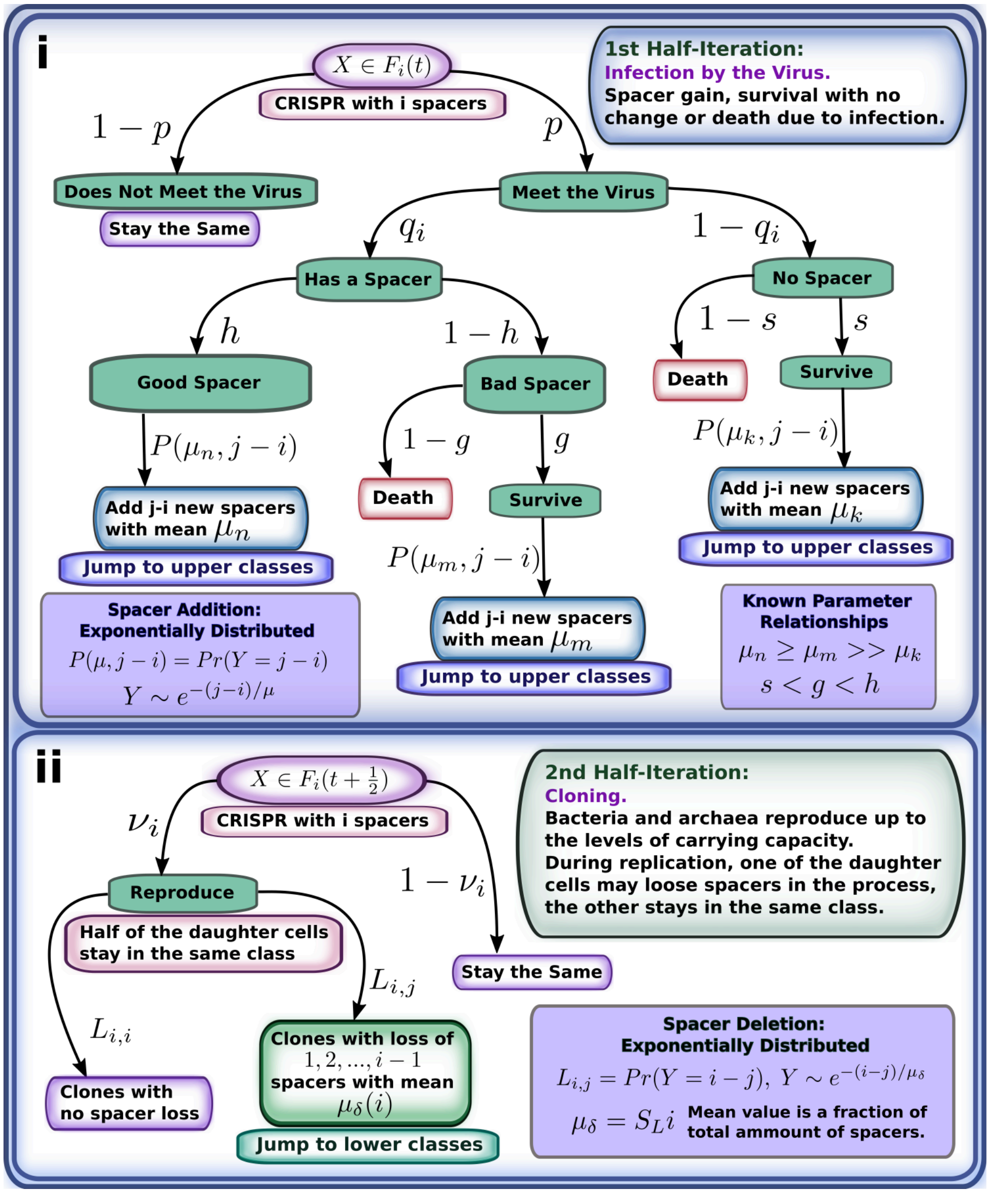

Fig A. Flowchart of the mathematical model presenting possible outcomes of bacteria-phage interaction and replication for bacteria having i spacers. (**i**) 1st half-iteration describes infection by the phage resulting in spacer gain or bacterial death; (**ii**) 2nd half-iteration includes the replication of bacteria which can result in spacer loss. The parameters of the model are discussed in the text.

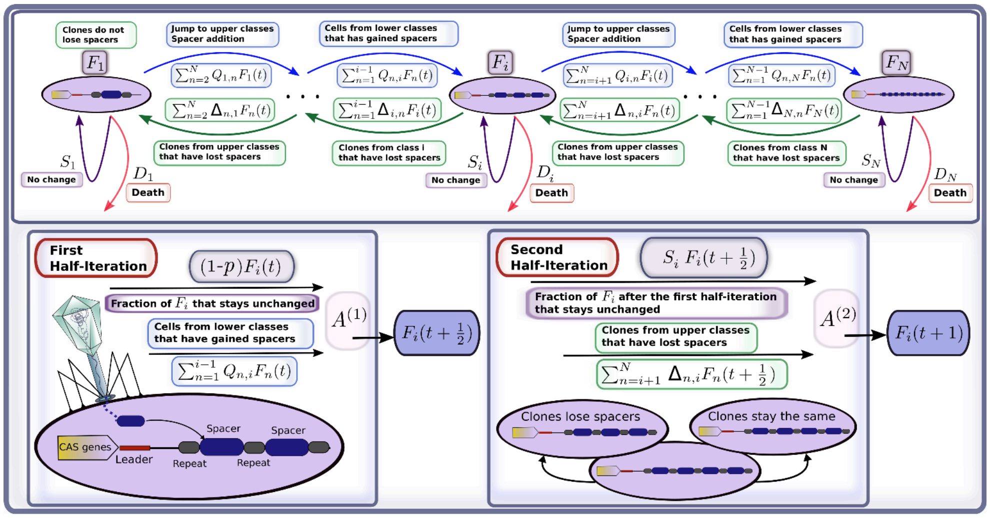

Fig B. Schematic representation of transition between size classes (containing different spacer numbers i) at time iteration t. The first half-iteration describes the infection in the case the cell meets a phage. Possible outcomes of this stage are: gaining $j - i$ spacers (with probability $Q_{i,j}$), death of the cell, and survival without gaining spacers (with probability $1 - p$). The second half-iteration mimics the replication stage. Replication can result in loss of $i - j$ spacers in the daughter cell. The parent cell is assumed to always keep the same number of spacers. The loss or conservation of spacers number at this stage are described, respectively, by $\Delta_{j,i}$ and S_i.

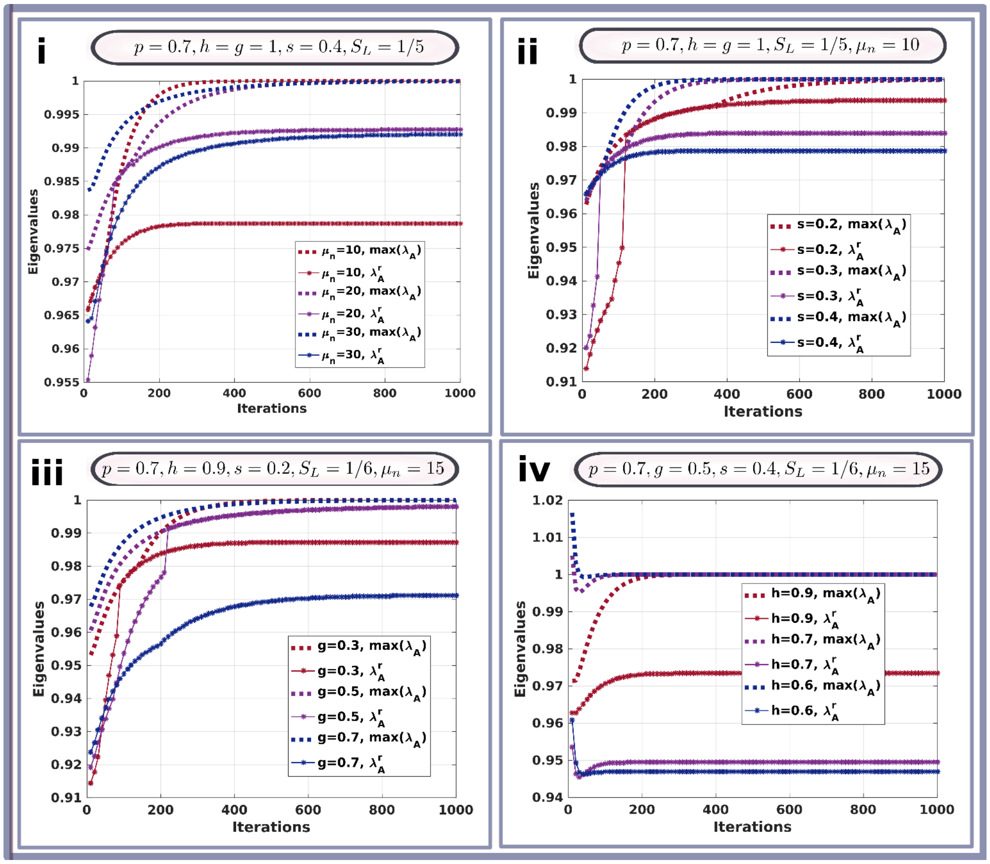

Fig C. Temporal evolution of the transition matrix eigenvalues. Here $\max(\lambda_{\mathbf{i}})$ is the largest eigenvalue of the transition matrix $\mathbf{i} = \mathbf{i}^{(2)}\mathbf{i}^{(1)}$, $\lambda_{\mathbf{i}}^r$ is the second eigenvalue determining the rate of convergence. Convergence rate is determined by the dynamics of maximal eigenvalues which are plotted for different model parameters: (**i**) $\mu_n = 10, 20, 30$, (**ii**) $s = 0.2, 0.3, 0.4$, (**iii**) $g = 0.3, 0.5, 0.7$, (**iv**) $h = 0.9, 0.7, 0.6$. $\max(\lambda_{\mathbf{i}}) \to 1$ as the number of iterations gradually increases.

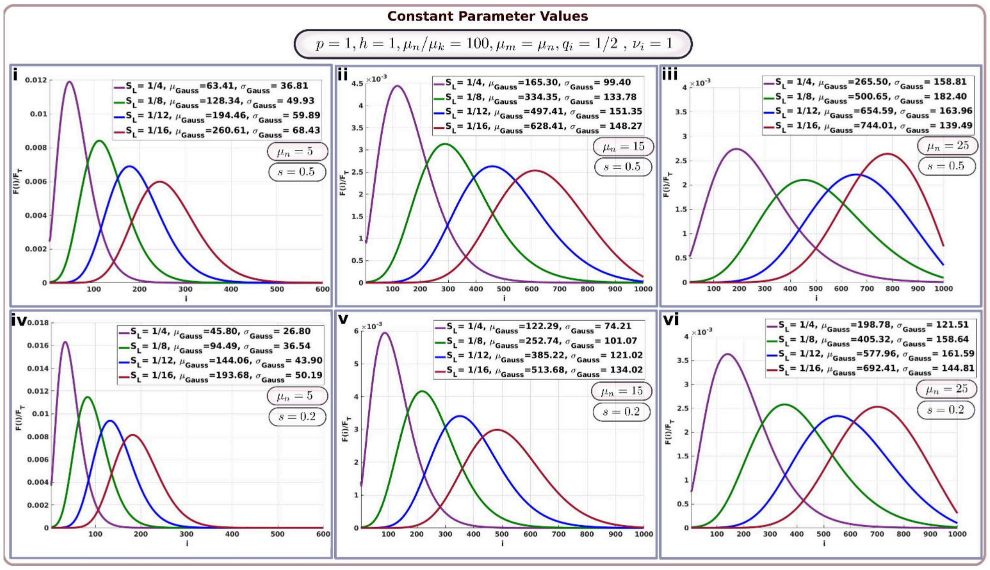

Fig D. Statistical distribution of spacers in CRISPR array predicted by the model with constant q_i and ν_i. Here $q_i = \frac{1}{2}$ and $\nu_i = 1$ for all i. The resultant stationary distribution is of Gaussian type. From numerical simulation, the mean value can be estimated as $\mu_{Gauss} = \left(\sum_{i=1}^{N} iF_i\right)/F_T$ and the variance $\sigma^2_{Gauss} \approx \frac{1}{F_T}\sum_{i=1}^{N} F_i(i - \mu_{Gauss})^2$. (**i**, **ii**, **iii**) $s = 0.5$, (**iv**, **v**, **vi**) $s = 0.2$. Graphs show normalized class sizes F_i/F_T after 1000 iterations from the uniform initial condition $F_i(0) = 1000$ and maximum spacer number $N = 1000$. The population size is $F_T = 10^6$. Keeping other parameters fixed, increasing μ_n or decreasing the fraction of spacers lost during the replication S_L shifts μ_{Gauss} to the right. The variance σ^2_{Gauss} increases with decreasing S_L and increasing μ_n, provided the Gaussian mean $\mu_{Gauss} < N/2 = 500$ (**i**, **ii**, **iv**, **v**). Smaller values of s tend to decrease the variance and the Gaussian mean, but also only for moderate values of μ_n, where the distribution is centered more towards the left.

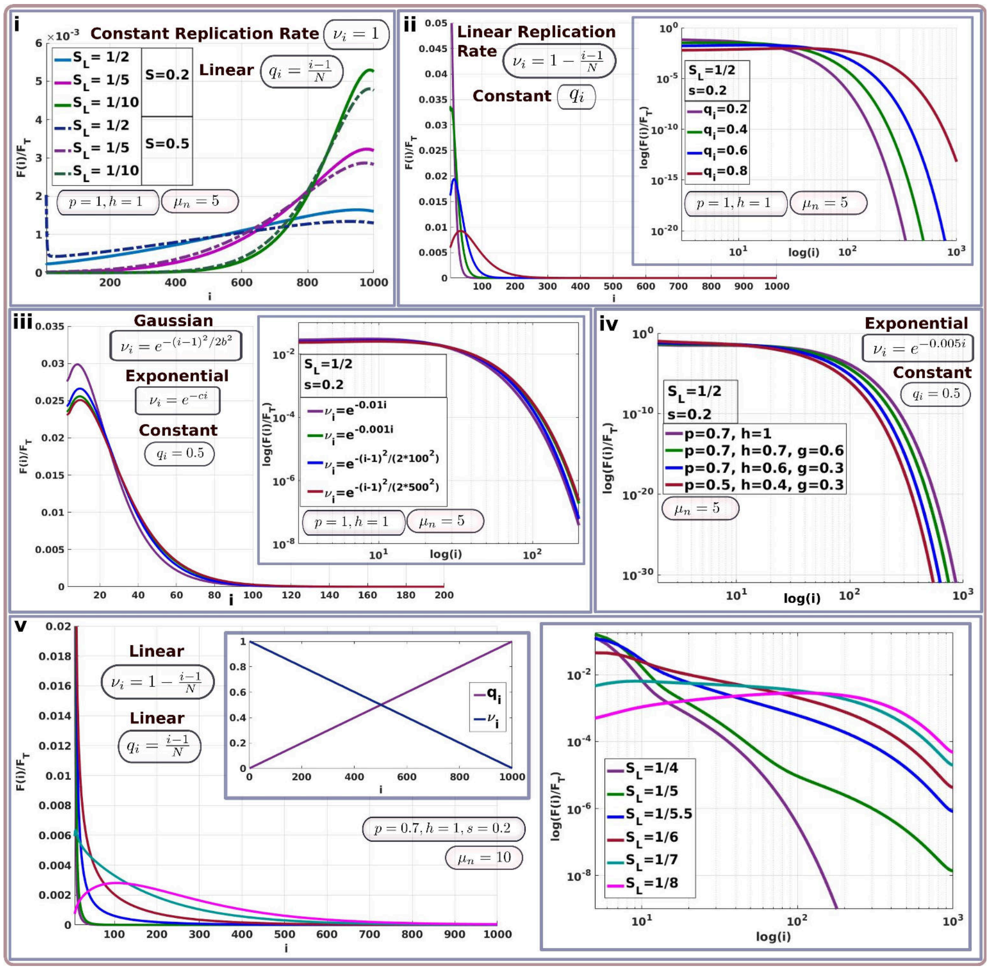

Fig E. Introducing parameter dependence on the CRISPR array length i. (**i**) For a constant replication rate ν_i and linear increasing $q_i = \frac{i-1}{N}$, larger values of μ_n and smaller values of S_L increase the right-centeredness of the distribution. Decreasing μ_n to 5 and increasing S_L to 1/2 did not produce the desired left-centered distribution. Decreasing the value of s generally tends to amplify the right or left-centeredness. (**ii**) For a constant probability of having a spacer for the virus q_i and linear decreasing ν_i, the distribution is left-centered; however this is not a heavy-tailed one (the right figure shows the characteristic normal distribution ($\mu_{Gauss} \approx 0$) log-log plot. Using exponentially decaying and Gaussian replication rates (**iii**) as well as changing other constant parameter values p, h, s, and g (**iv**) do not generate any difference away from the normal distribution type. Decrease in constant probability values in (**iv**) tend to decrease the variance of the resulting distribution. (**v**) In the case, where we consider q_i and ν_i to be both linear functions, by varying S_L we can finally observe a heavy-tailed distribution. In the case, $\mu_n = 10$, $s = 0.2$, $p = 0.7$, $h = 1$ a fat tail distribution is observed in the range of $1/7 < S_L < 1/4$. Values of $S_L \leq 1/7$ do not provide the required left-centeredness (not enough microbes with spacer loss get added to lower classes) and for values of S_L too large, almost all cells end up in class F_1 (too much spacer loss, not enough spacer gain).

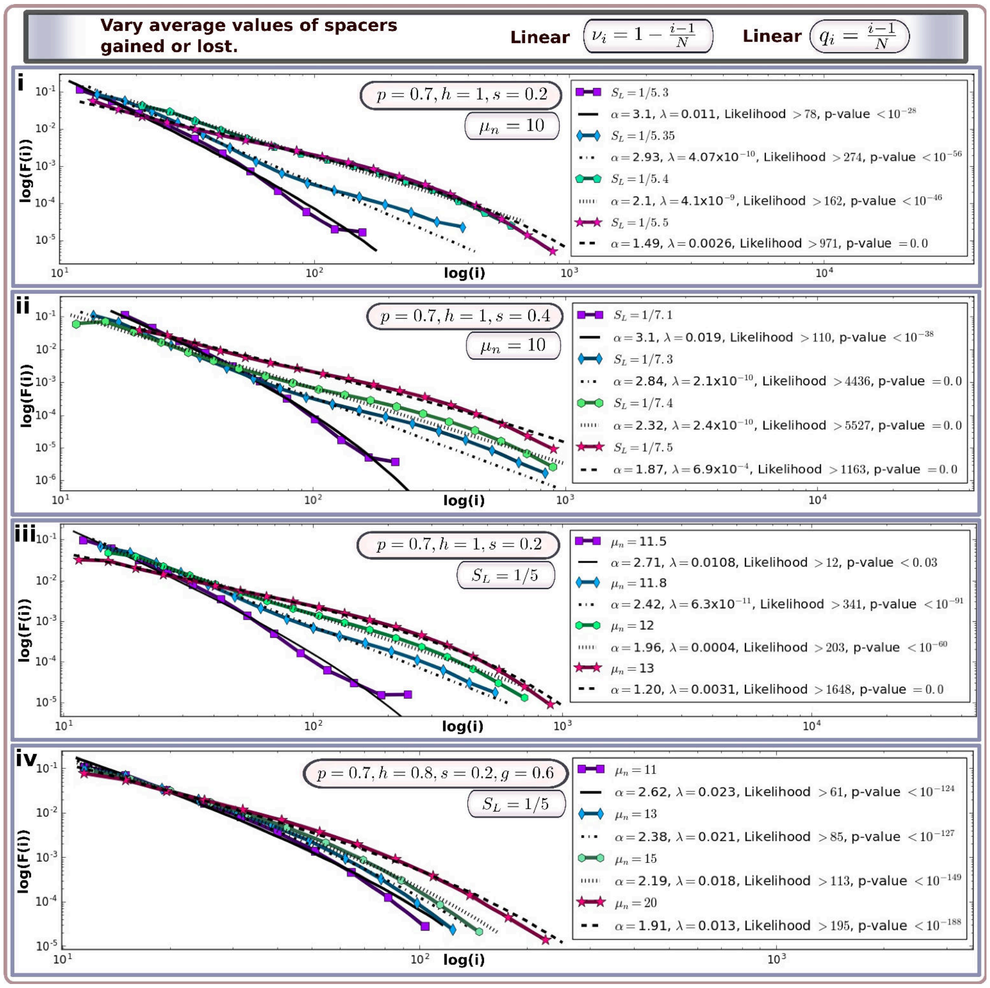

Fig F. Spacer distribution for linear dependence of q_i and ν_i affected by variation of S_l and μ_n. Effects of variation of S_L for $s = 0.2$ (**i**) and $s = 0.4$ (**ii**). The other parameters are fixed at $p = 0.7$, $h = 1$, $100 * \mu_k = \mu_n = \mu_m = 10$. The best fit option is a truncated power law (as compared to lognormal, exponential, and stretched exponential distributions). The minimum livelihood and maximum p-values for all comparison cases are labeled in the figure legend. In both cases ($s = 0.2, 0.4$), decreasing S_L reduces the value of α. Effects of variation of μ_n for $h = 1$ (**iii**) and $h = 0.8$, $g = 0.6$ (**iv**). The other parameters are $p = 0.7$, $s = 0.2$, $S_L = 1/5$. The found best fit is provided by a truncated power law. Increasing μ_n generally works to decrease the power law exponent α and to increase the range of nonempty classes F_i (microbes are able to accumulate more spacers).

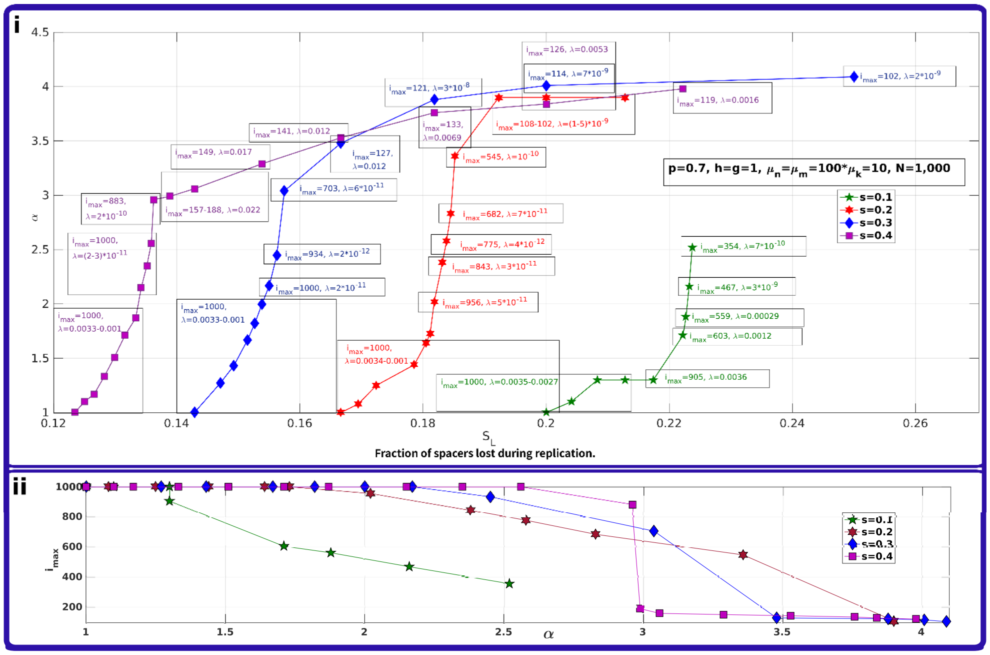

Fig G. Sensitivity of α with respect to variation of S_L (the fraction of spacers lost during replication) and s (the probability of survival if the microbe has no spacer for the virus). (**i**) For the fixed parameter values $p = 0.7$, $h = 1$, $100 * \mu_k = \mu_n = \mu_m = 10$ and $s = 0.1, 0.2, 0.3, 0.4$ the values of S_L that gave livelihood values > 100 and $i_{max} > 100$ are shown in the figure. The truncated power law fit is performed with $i_{min} \leq 15$ and i_{max} set to the data maximum. The p-values for the truncated power law fit (as compared to other distributions) in all cases do not exceed 10^{-9}. Increasing S_L above the maximum values shown on the graph result in low maximum numbers of spacers $i_{max} < 100$ and small or negative likelihood values. Decreasing S_L result in lowering the slope of the distribution, from the maximum α obtained at the maximum admissible S_L towards the minimum $\alpha = 1.00$. Decrease in S_L past the minimum values shown on the graph result in negative likelihood values. For smaller s, the fraction of spacers lost during replication S_L has to be larger in order to generate equivalent power law behavior characterized by the same exponent α. (**ii**) Decrease in α within an intermediate range is proportional to an increase in i_{max}, with $i_{max} = N = 1000$ (the maximum number of spacers) for $\alpha = 1.00$. Larger values of s yields an increased range of α with the maximum number of nonempty classes $i_{max} = N = 1000$.

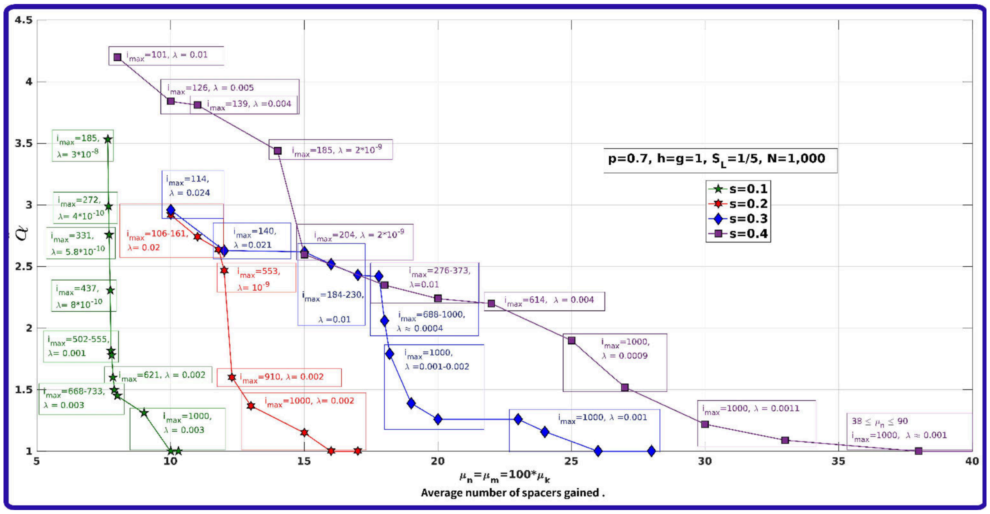

Fig H. Sensitivity of α with respect to variation of μ_n (the average number of spacers gained) and s (the probability of survival if the microbe has no spacer for the virus). For the fixed parameter values $p = 0.7$, $h = 1$, $S_L = 1/5$, $100 * \mu_k = \mu_n$ and $s = 0.1, 0.2, 0.3, 0.4$ the values of μ_n with the likelihood values > 100 and $i_{max} > 100$ are shown in the figure. The truncated power law fit is performed with $i_{min} \leq 15$ and i_{max} set to the data maximum. The p-values for the truncated power law fit (as compared to other distributions) in all cases do not exceed 10^{-9}. Decreasing μ_n below the minimum values shown on the graph result in low maximum numbers of spacers $i_{max} < 100$ and small or negative likelihood values. Increasing μ_n result in decreasing the slope of the distribution, from the maximum α obtained at the minimum admissible μ_n towards the minimum $\alpha = 1.00$. Decrease in α through intermediate ranges is proportional to the increase in i_{max} with $i_{max} = N = 1000$ (the maximum number of spacers) for $\alpha = 1.00$. Increase in μ_n past the maximum values shown on the graph result in negative likelihood values. Larger values of s yield an increased range of α with the maximum number of nonempty classes $i_{max} = N = 1000$ and a larger range of μ_n that generate a desired fit. For $s = 0.4$, the truncated power law fit with $\alpha = 1.00$, $\lambda = 0.001$ and the likelihood > 1000 is observed for $30 \leq \mu_n \leq 90$, while for $s = 0.1$ the maximum admissible μ_n is less than 10.4.

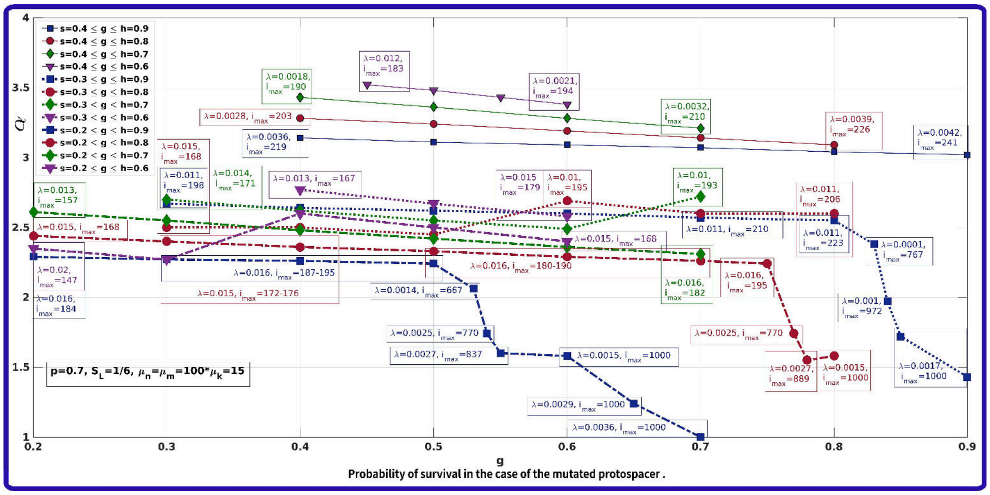

Fig I. Sensitivity of α with respect to variation of $h < 1$ (the probability of protospacer match), g (the probability of survival in the case of mutated protospacer) and s. $s \leq g \leq h$. For the fixed parameter values $p = 0.7$, $S_L = 1/6$, $100 * \mu_k = \mu_n = \mu_m = 15$, $s = 0.4, 0.3, 0.2$ and $h = 0.9, 0.8, 0.7, 0.6$, the values of g in the interval between s and h that produce likelihood values > 100 and $i_{max} > 100$ are shown in the figure. The truncated power law fit is performed with $i_{min} \leq 15$ and i_{max} set to the data maximum. The p-values for the truncated power law fit (as compared to other distributions) in all cases do not exceed 10^{-9}. Larger values of s and smaller values of h result in larger α in all cases. The value of α is generally non-increasing as g is increased from s to h. For larger values of s, α does not change significantly throughout the interval.

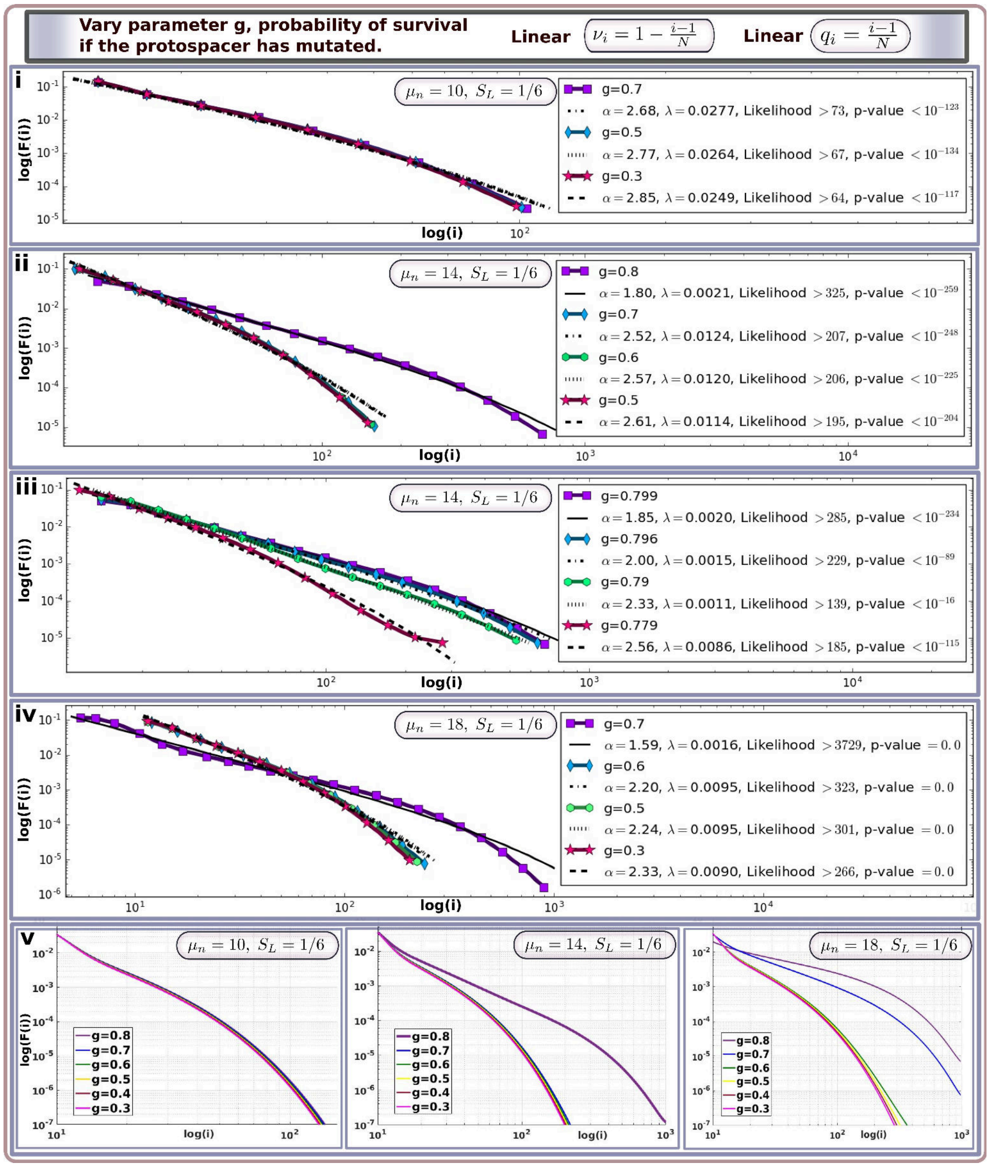

Fig J. Variation of statistical distribution of spacers (linear q_i and ν_i) for different g (the probability of survival in the case of mutated protospacer). For the fixed parameter values $p = 0.7$, $s = 0.2$, $h = 0.8$, $100 * \mu_k = \mu_m = \mu_n$, g is reduced sequentially in the interval $h \leq g < s$. (**i**) With fixed parameter values $S_L = 1/6$ and $\mu_n = 10$, decreasing g from 0.8 to 0.3 does not produce a significant change in power law parameter values. A small increase in α is mainly due to the fitting algorithm choice of i_{min}. An increase $\mu_n = 14$ (**ii**, **iii**) and decrease in g from 0.77 to 0.3 also produces only very slight increase in α from 2.5 to 2.6. However, the rate of increase of α is significantly faster for $0.8 \leq g \leq 0.77$ (**iii**). The range of non empty classes follows the same pattern, for $0.8 < g < 0.78$, i_{max} it decreases from 771 to 300 and with a further decrease in g, i_{max} it stays around 160. (**iv**) Taking even larger value of $\mu_n = 18$, the interval where α decreases fast with decreasing g is shifted towards lower values $0.7 < g < 0.6$. In that region we can again observe large i_{max} values. (**v**) Results of numerical simulation (MATLAB) for stationary class sizes that were analyzed using python powerlaw package (**i-iv**).

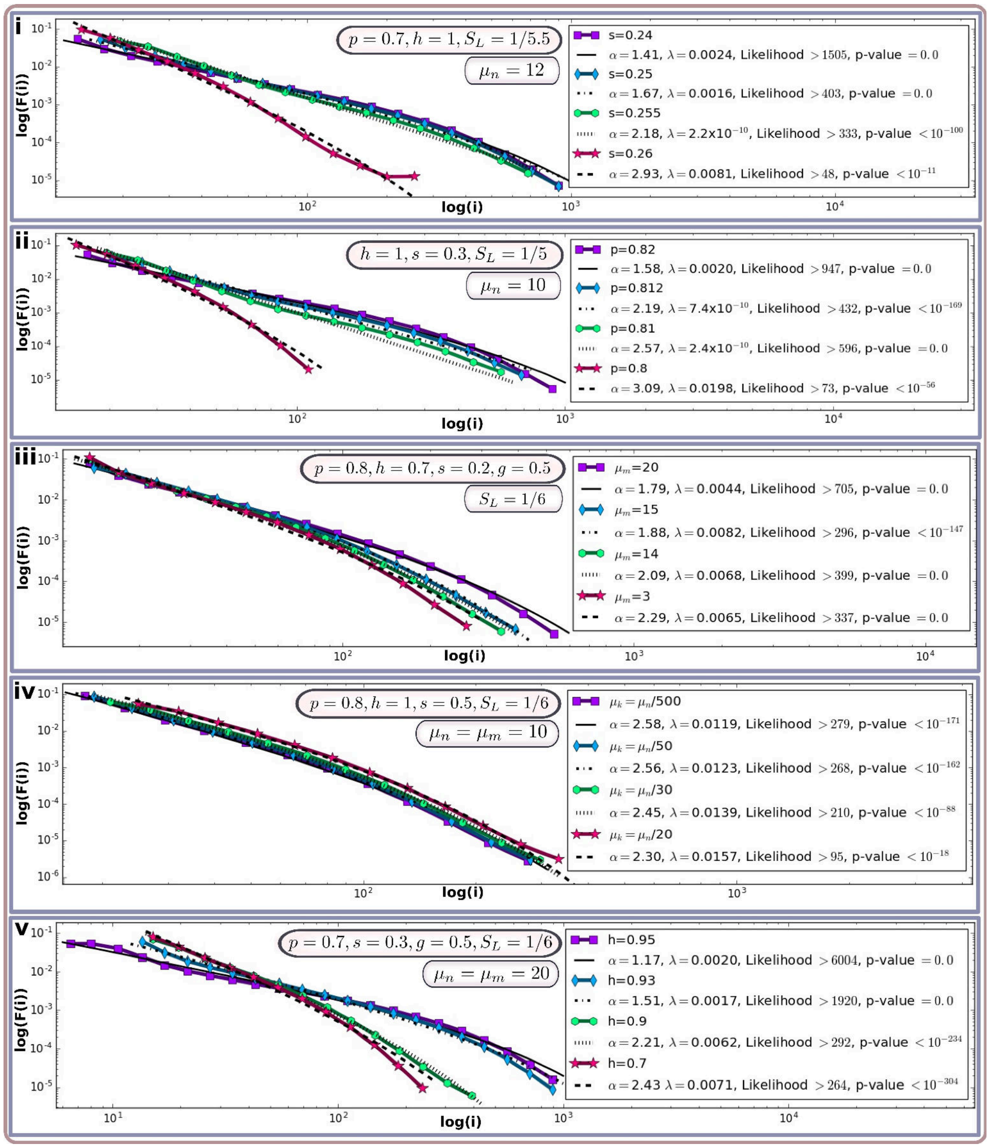

Fig K. Variation of statistical distribution of spacers (linear q_i and ν_i) for different parameters s, p, μ_m, μ_k and h. (**i**) For the fixed parameter values $p = 0.7$, $h = 1$, $S_L = 1/5.5$, $100 * \mu_k = \mu_m = \mu_n = 12$, as s is increased sequentially from 0.24 to 0.26 α increases from 1.141 to 2.9. (**ii**) For the fixed parameter values $h = 1$, $s = 0.3$, $S_L = 1/5$, $100 * \mu_k = \mu_m = \mu_n = 10$, decreasing p from 0.82 to 0.8 α increases from 1.58 to 3.09. Further decrease in p does not generate significant changes in α, $\alpha \approx 3.1$ for all values of $p < 0.8$. (**iii**) For the fixed parameter values $p = 0.8$, $h = 0.7$, $s = 0.2$, $g = 0.5$, $S_L = 1/6$, $100 * \mu_k = \mu_n = 20$, decreasing μ_m below μ_n works to increase α. (**iv**) For the fixed parameter values $p = 0.8$, $h = 1$, $s = 0.5$, $S_L = 1/6$ and $\mu_n = \mu_n = 10$, increasing μ_k in relation to μ_n decreases α only slightly. As μ_k changes from $\mu_n/500$ to $\mu_n/20$, α decreases from 2.58 to 2.3.(**v**) For the fixed parameter values $p = 0.7$, $g = 0.5$, $s = 0.3$, $S_L = 1/6$ and $100 * \mu_k = \mu_n = \mu_n = 20$, decreasing h from 0.95 to 0.9 increases α from 1.16 to 2.2, further decrease on h does not generate significant changes, $\alpha \approx 2.3$.
